# Rapid Expansion of a Highly Germline-Expressed *Mariner* Element Acquired by Horizontal Transfer in the Fire Ant Genome

**DOI:** 10.1093/gbe/evy220

**Published:** 2018-10-09

**Authors:** Chih-Chi Lee, John Wang

**Affiliations:** 1 Biodiversity Research Center, Academia Sinica, Taipei, Taiwan; 2 Laboratory of Insect Ecology, Division of Applied Biosciences, Graduate School of Agriculture, Kyoto University, Japan; 3 Research Institute for Sustainable Humanosphere, Kyoto University, Kyoto, Japan

**Keywords:** transposable elements, fire ants, germline transcriptome, horizontal transfer

## Abstract

Transposable elements (TEs) are present in almost all organisms and affect the host in various ways. TE activity can increase genomic variation and thereby affect host evolution. Currently active TEs are particularly interesting because they are likely generating new genomic diversity. These active TEs have been poorly studied outside of model organisms. In this study, we aimed to identify currently active TEs of a notorious invasive species, the red imported fire ant *Solenopsis invicta*. Using RNA profiling of male and female germline tissues, we found that the majority of TE-containing transcripts in the fire ant germline belong to the *IS630-Tc1-Mariner* superfamily. Subsequent genomic characterization of fire ant *mariner* content, molecular evolution analysis, and population comparisons revealed a highly expressed and highly polymorphic *mariner* element that is rapidly expanding in the fire ant genome. Additionally, using comparative genomics of multiple insect species we showed that this *mariner* has undergone several recent horizontal transfer events (<5.1 My). Our results document a rare case of a currently active TE originating from horizontal transfer.

## Introduction

Transposable elements (TEs) are parasitic genetic elements that can jump to different positions in the genome and, occasionally, into other genomes. Virtually all organisms harbor TEs, with genome occupancy ranging from <1% to ∼85% in multicellular animals and plants (Lander et al. 2001; Schnable et al. 2009; Maumus et al. 2015).

TE insertions are typically deleterious or neutral in animals and plants. Although infrequent, many instances of adaptive insertions have been documented (Aminetzach 2005; Rostant et al. 2012; Mateo et al. 2014; Hof et al. 2016). TEs can also induce different types of chromosomal rearrangements through ectopic recombination that can modify or even delete genes (Warren et al. 2015). Therefore, TEs can play an important role in the evolution of host fitness and genome structure.

The life cycle of a TE begins upon entering a host and acquiring activity in the germline (Huang et al. 2012). Often, TEs may undergo in an initial proliferative “burst,” or rapid increase in copy number, before the host defense system suppresses the new invader through small RNA pathways (e.g., piRNAs, miRNAs, and siRNAs) and chromatin remodeling via histone and DNA methylation (Pinsker et al. 2001; Friedli and Trono 2015; Fultz et al. 2015), although exceptions may exist. After host defenses have adapted, however, leaky transposition events may continue to occur (Scott et al. 2016). TE self-regulation mechanisms may also evolve (Kidwell and Lisch 2001; Feschotte and Pritham 2007; Bire et al. 2016). Eventually, the ultimate fate of a TE inside a host, aside from rare domestication events, is loss through silencing and degeneration (Brookfield 2005). Consequently, the long-term survival of a TE must be through horizontal transposon transfer (HTT) (Feschotte and Pritham 2007; Schaack et al. 2010). On the evolutionary timescale, HTT between eukaryotic species (and between prokaryotic species) is a common and widespread phenomenon that can be viewed as starting a new life cycle for the TE (Muñoz-López and García-Pérez 2010; Venner et al. 2017).

Currently active TEs are particularly interesting because they are likely generating new genomic diversity, and thereby occasionally promoting adaptation. The rice *mPing* element provides a potentially illustrative case. This TE is currently increasing by ∼40 inserts per generation in some landraces, and such landraces are more stress resistant (Naito et al. 2009).

To be active, an autonomous TE must be transcriptionally expressed in the cells or progenitors of the germline. Thus, germline expression analysis may reveal candidate currently active TEs. In animals, this could be done by profiling gene expression from dissected gonads, which is simpler than purifying germ cells. Expressed TEs would need to be filtered by additional criteria (e.g., the presence of many identical copies and population polymorphism) (Mills et al. 2007) because some may be domesticated (Warren et al. 2015) or derived from the surrounding somatic support tissues of the ovaries or testes.

We are interested in how active TEs might be shaping the evolution of a social insect, the red imported fire ant *Solenopsis invicta*. This species has established itself in the United States, Australia, China, and Taiwan (Ascunce et al. 2011), and recently has been discovered in Japan and South Korea (Ujiyama and Tsuji 2018). As a major pest species of important economic and ecological impact, *S. invicta* has been extensively studied (Tschinkel 2006); this includes the development of genetic and genomic tools developed for this species (Wang et al. 2007, 2008; Lu et al. 2009; Wurm et al. 2011; Choi et al. 2012). One particularly interesting aspect of their biology is that a social supergene, composed of multiple inversions, regulates whether colonies have only one or many queens (Keller and Ross 1998; Gotzek and Ross 2007; Wang et al. 2013; Huang and Wang 2014; Huang et al. 2018). Notably, the polygyne colony-specific *Sb* allele of the supergene has accumulated TEs over evolutionary time (Wang et al. 2013). Aside from this, only a few other studies have examined TE content in *S. invicta* (Krieger and Ross 2003; Wang et al. 2008; Oliveira et al. 2012) or any other invasive species, either solitary or social (Schrader et al. 2014; Goubert et al. 2017), and none have examined TE activity.

In this study, we examined currently active TEs in *S. invicta*. Based on the life history of a TE, we hypothesized that currently active TEs could be found via RNA profiling of the germline and would exhibit copy number expansion after HTT. Such highly expressed TEs could potentially generate genomic diversity. Here we report the identification of such an element, using a combination of RNA-sequencing (RNA-seq), genomic characterization of TE content, molecular evolution analysis, population comparisons, and comparative genomics. We found that one *mariner* element has been and is likely currently active. Furthermore, this element has undergone several recent horizontal transfer events and has continued proliferating in the fire ant. This is the first identification of a currently active TE via RNA profiling in a social or an invasive species.

## Materials and Methods

### Sample Collection and Social Form Determination

We collected both monogyne and polygyne colonies of the red imported fire ant in Taoyuan City, Taiwan in October 2012. Ant colonies were returned to the lab and raised under standard conditions (Jouvenaz et al. 1977). We determined the social form of each colony based on an initial observation of a single, large physogastric queen (monogyne) or many smaller queens (polygyne) and subsequent confirmation by genotyping 10–13 workers using a PCR-RFLP assay for the *Gp-9* gene (Krieger and Ross 2002). The *Gp-9* gene is a marker for the social chromosome pair that underlies the two fire ant social forms (Wang et al. 2013).

### RNA Isolation and RNA-Seq Library Preparation

To identify TEs expressed in the germline, we extracted RNA from fire ant ovaries and testes. To obtain “mature” ovaries for RNA isolation, we artificially removed the wings from randomly selected virgin queens to stimulate ovary development. We took this approach because fire ant virgin queens usually initiate reproductive development after shedding their wings under natural conditions when no reproductive queen is present (Fletcher et al. 1983; Vargo and Fletcher 1986; Burns et al. 2007; Wurm et al. 2010), and artificial removal of wings in other ants induces ovary development (Jemielity et al. 2006). Polygyne virgin queens can be of two predominant social chromosome genotypes (*SB/SB* or *SB/Sb*) (Wang et al. 2013), and thus for these virgin queens, we isolated DNA from the removed wings (QuickExtract DNA Extraction Solution [Epicentre]) and genotyped them at the *Gp-9* locus (Krieger and Ross 2002). To ensure that the ovaries were mature, we dissected virgin queen ovaries seven days after the first egg was laid. In total, we sampled 10 and 11 pairs of ovaries from *SB/SB* monogyne (monogyne ovary, MO; derived from one monogyne colony) and *SB/Sb* polygyne virgin queens (polygyne ovary, PO; derived from one polygyne colony), respectively.

Because the testes degenerate in the adult stage and since germ cells are the ones contributing to all future generations (Haig 2016; Tóth et al. 2016), we decided to dissect testes from presumptive third and fourth instar larvae (body length > 3 mm; *n* = 63, all from one colony). At the time of the experiment, we did not have males from polygyne colonies, so we used only *SB* males from monogyne colonies (monogyne testis [MT]).

We purified total RNA from these germline tissues with the Illustra RNAspin Mini Kit (GE Healthcare Life Sciences). Total RNA was then submitted to the High Throughput Genomics Core at the BRCAS (Taipei, Taiwan) who enriched for mRNA by polyA selection. After, polyA-enriched RNA was sequenced on the Illumina HiSeq-2500 and Genome Analyzer platforms, with paired-end read lengths of 101 bp and 96 bp, respectively. In total, we obtained 4.8 Gb (MO), 5.0 Gb (PO), and 5.1 Gb (MT) of RNA sequence data.

### Sequence Assembly of the Germline Transcriptomes

We assessed the quality of the raw RNA-seq reads with FastQC v0.11.5 (Andrews 2010), which revealed that the first 15–18 nts at the 5′ end were nonrandom (18 nts in MO, 17 nts in PO, and 15 nts in MT). Thus, we trimmed these respective sequences with cutadapt v1.9.1 (Martin 2011) using the paired-end mode and also filtering against reads with less than 50 nts (after trimming), 3′ Illumina adapter sequence, and low-quality bases (-a <adapter sequence> -q 28 -m 50 –cut 18; adapters were Illumina TruSeq adapter index 4 [MO], index 2 [PO], and index 5 [MT]). We conducted de novo assembly of the three germline transcriptomes separately using Trinity v2.2.0 (Grabherr et al. 2011) with a minimum contig length of 305 bp (–min_contig_length 305; other parameters, default).

We assessed the quality of the assemblies in three ways. First, for each assembly we determined the percentage of all paired reads that mapped back to the assembly, and of these, how many were proper pairs. To do this we aligned reads using Bowtie2 v2.2.1 (Langmead and Salzberg 2012), and calculated alignment statistics using the script SAM_nameSorted_to_uniq_count_stats.pl (https://github.com/trinityrnaseq/trinityrnaseq/wiki/RNA-Seq-Read-Representation-by-Trinity-Assembly; last accessed September 21, 2016). Second, we evaluated the completeness of the assembled genes by comparing each transcriptome assembly to the predicted proteins in the fire ant official gene set (GCF_000188075.1_Si_gnG_protein.faa downloaded from NCBI) using analyze_blastPlus_topHit_coverage.pl. For each gene, we considered the assembly adequate if the top BLASTx transcript hit covered ≥80% of the protein length. Finally, we determined the representativeness of 2,675 conserved single-copy orthologs in the arthropod lineage (BUSCOv1.22 arthropod sequences downloaded September 2016; command: python BUSCO_v1.22.py -m trans -l arthropoda) (Simão et al. 2015).

### Identification of Highly-Expressed Autonomous Transposable Elements in the Germline

To annotate TE-derived sequences in transcripts from the fire ant germline, we used RepeatMasker open-4.0.6 (Smit et al. 2014) and the RMBlast algorithm to compare the three germline transcriptomes against the TE database from Repbase v20.09 (Bao et al. 2015), which includes 171 described TEs in *S. invicta*. We retained the TEs with masked length ≥200 bp and divergence ≤20%. These criteria should identify germline TEs based on sufficient similarity regardless of annotation state in Repbase and without too many false positives. A caveat is that we may fail to include novel or highly divergent TEs. For estimating transcript expression levels, we first removed rRNA sequences with SortMeRNA (Kopylova et al. 2012) and then calculated TPM (transcripts per million) values at the gene level (i.e., not isoform) using RSEM (via Trinity scripts, see supplementary materials), an alignment-base quantification method that uses a statistical model to handle reads with multiple hits (Li et al. 2010; Li and Dewey 2011). We defined “highly expressed” TE candidates as those transcripts with TPM values greater than that of 75% of the single-copy BUSCO genes that could be annotated in the germline transcriptomes (i.e., 1,809 BUSCO genes in MO, 1,812 in PO, and 1,747 in MT). We also estimated TE expression levels using Repbase v20.09 (Bao et al. 2015) as the reference in a similar manner, except we only considered the top 20 expressed TEs in each of the three germline data sets.

For all highly expressed candidate TE transcripts, we conducted the following additional analyses. As autonomous TEs should contain an intact transposase, we used the getorf program from the EMBOSS 6.6.0 package (Rice et al. 2000) to identify intact ORF’s (-find 3 -minsize 300) and used tBLASTx (-evalue 1e-20) (Camacho et al. 2009) to query against the TE database from Repbase v20.09 (Bao et al. 2015). To exclude possible contamination from other organisms, we manually verified that each TE was present in the fire ant genome (both published and our unpublished versions). Finally, for 17 candidates with a detectable transposase or reverse transcriptase open reading frame (RT-ORF), we reconstructed the consensus TE sequence as follows. First, we extracted candidate TE reference sequences from Repbase v20.09 (Bao et al. 2015). We then used BLASTn (Camacho et al. 2009) to query these TEs against the fire ant reference genome to obtain a set of all TE copies. Next, we generated the respective fire ant TE consensus sequences by extracting genome sequence with hit length ≥100 bp and assembling them with CAP3 (Huang and Madan 1999). Finally, we confirmed the TE structure. Terminal inverted repeats (TIR) were identified with the einverted program from the EMBOSS 6.6.0 package (Rice et al. 2000) and long terminal repeats (LTR) for RNA TEs were identified with REPFIND (Betley et al. 2002). We further confirmed that the intact ORF was a transposase by conducting a SmartBlast query against the UniProtKB/Swiss-Prot database in NCBI ORF finder.

### Investigation of Active Transpositions in the Fire Ant Genome

To detected de novo and polymorphic TE insertions we used a previously generated data set comprising of seven pairs of *SB* and *Sb* brothers derived from different *SB/Sb* queens collected from Georgia, USA. These haploid males were sequenced at ∼6–8× coverage (SRA Study accession SRP017317) (Wang et al. 2013). Quality assessment of the raw reads with FastQC v0.11.5 (Andrews 2010) revealed that the first 2–10 nts (data sets differed) from the 5′ end were nonrandom. Thus, we trimmed these respective sequences with cutadapt v1.9.1 (Martin 2011) using the paired-end mode and filtering against reads with less than 50 nts, 3′ Illumina adapter sequence, and low-quality bases (-a <adapter seq> -q 28 -m 50).

Using this data set, de novo insertions can only be identified in regions where both brothers inherited the same allele from the mother, for example, regions identical by descent (IBD). To find IBD regions, we first determined the single-nucleotide polymorphism (SNP) densities at 10 kb intervals across the genome between each pair of brothers. Regions with very low SNP density would indicate genomic regions likely IBD. In contrast, genomic regions with high SNP density would indicate genomic regions where the brothers inherited different maternal alleles. Variant calling followed the GATK best practices (Van der Auwera et al. 2013). In brief, we mapped sequences to the reference with BWA (Li and Durbin 2010) and called variants within each family with GATK UnifiedGenotyper. Next, we removed homozygous SNPs (relative to the reference genome) and calculated SNP density in 10 kb windows with vcftools (Danecek et al. 2011).

To locate the de novo TE insertions, we used ngs_te_mapper (Linheiro and Bergman 2012). This program extracts raw reads containing TE sequences and then re-aligns this subset against the reference genome. De novo TE insertion sites are identified based on partially overlapping reads as a signature for target site duplications (TSD’s) which are generated upon TE insertion. We focused on 17 TEs highly expressed in the germline using our fire ant consensus TE sequences (above). To minimize the detection of false positives, we manually examined the BWA alignment maps for each candidate novel insertion. Read mapping scores <10 (mapq; probability of correct match <90%) were discarded. We excluded nonreference insertions in the *Sb* supergene region because we used *SB* as the reference genome.

For our reference genome we used a new version generated by Pacific Biosciences sequencing (assembly and annotation to be published separately, see supplementary table S1, Supplementary Material online for assembly metrics). We generated pseudochromosome scaffolds by placing and orienting the genome scaffolds using previous RAD-seq linkage data (Wang et al. 2013) with ALLMAPS (Tang et al. 2015). In total, we placed 236.64 Mb (67.8%) of the reference genome assembly into linkage groups. To reduce false positive variant calls due to repetitive sequences, we then masked the repetitive sequences using RepeatMasker open-4.0.6 (Smit et al. 2014) with the TE database from Repbase v20.09 (Bao et al. 2015) and masked tandem repeat sequences with Tandem Repeats Finder (Benson 1999). This masked genome was the reference genome assembly used for SNP calling and de novo TE insertion annotation.

To identify sets of identical TE sequences, we extracted the genomic sequence for all copies of the 17 TE consensus sequences in the fire ant genome (and also ≥60% full length) and then found redundancies among these copies using the script Sequence Dereplicator and Database Curator (SDDC) (Ibrahim et al. 2017).

We estimated TE copy number conservatively by counting only the number of BLASTn hits (coverage ≥60%) in the fire ant genome for the 17 query TE consensus sequences. We selected coverage ≥60% as the threshold because a large enough deletion within a gene may result in double counting with a lower threshold (i.e., a deletion in the middle may result in counting the remaining left and right fragments as separate hits). Additionally, this approach will underestimate the number of short miniature inverted-repeat transposable elements (MITEs).

To estimate mean genetic diversity (π) for each of the 17 TEs with high germline expression, we extracted all sequences with ≥60% of the full-length TE sequence from the fire ant genome. We then conducted sequence alignments with MUSCLE (Edgar 2004) in MEGA 7 (Kumar et al. 2016). Finally, we estimated π by the maximum composite likelihood method with uniform mutation rates among sites and 1,000 bootstrap replications in MEGA 7 (Kumar et al. 2016).

### Determination of *Mariner-2_DF* Distribution in Insects

To identify other insects having copies of *Mariner-2_DF*, we used BLASTn to query against the 52 arthropod genomes in Flybase (supplementary table S1, Supplementary Material online) (Attrill et al. 2016). We considered *Mariner-2_DF* to be present in the genome assembly using established criteria (Gilbert et al. 2010; El Baidouri et al. 2014; Zhang et al. 2014): at least one copy with hit length >100 bp and identity >90%.

### Consensus Sequence and Copy Number of *Mariner-2_DF* in Insects

For the reconstruction of the *Mariner-2_DF* consensus sequence in each species, we first downloaded the draft genome assembly for each of the eight species with *Mariner-2_DF*-like sequences from NCBI (supplementary table S1, Supplementary Material online). We reconstructed the species-specific consensus sequences as above for *S. invicta*. The ORF of each consensus sequence was extracted by the getorf program (-find 3). We used BLASTn and BLASTp (Camacho et al. 2009) to query the consensus sequences against each other to obtain pairwise nucleotide and amino acid identities.

To estimate *Mariner-2_DF* copy number in each genome, we used BLASTn (Camacho et al. 2009) to query each species-specific consensus sequences against the corresponding genome assembly. Because TEs and other repetitive sequences prevent full genome assembly (Treangen and Salzberg 2012), many TE copies in the genome are partial. For this study, we only considered elements with at least 40% coverage of the consensus sequence in estimating copy number (Zhang et al. 2014). Specifically, since the full length of *Mariner-2_DF* is 1,324 bp, all hits ≥528 bp from the BLASTn results were counted. Still, this approach will underestimate the number of MITEs that might be derived from *Mariner-2_DF*.

### Estimation of Synonymous Substitution Rates (Ks)

To estimate the synonymous substitution rates (Ks) between species, we extracted *Mariner-2_DF* ORFs used the getorf program (-find 3 -minsize 1059) from the EMBOSS 6.6.0 package (Rice et al. 2000). For *D. ficusphila* and *A. echinatior*, which lack intact transposase ORFs (due to mutations) in the genome, we used BLASTn hits ≥1,059 bp (the full length of the transposase ORF) instead. We aligned sequences based on amino acids using MUSCLE (Edgar 2004) and selected conserved codon blocks with Gblocks v0.91b (Castresana 2000; Talavera and Castresana 2007). We conducted interspecies pairwise comparisons. For example, there were 14 *Mariner-2_DF* copies from *D. grimshawi* and 281 copies from *S. invicta*. Thus, we computed 14 × 281 = 3,934 Ks values in the *D. grimshawi* and *S. invicta* pair.

To compare Ks values from *Mariner-2_DF* with those from nuclear genes, we downloaded the transcriptome of each insect from NCBI (supplementary table S1, Supplementary Material online). We extracted intact ORFs from the RNA transcriptome with the getorf program (-find 3). For each species, we identified the orthologous genes that corresponded to the 2,675 BUSCOv1.22 arthropod gene set (downloaded September 2016; command: python BUSCO_v1.22.py -m trans -l arthropoda) (Simão et al. 2015). Then, we selected the intersection, 1,951 genes, to estimate Ks values. After, we aligned nucleotide sequences pairwise based on the amino acids in MUSCLE (Edgar 2004) and removed gaps used PAL2NAL (Suyama et al. 2006).

We used the KaKs_calculator 2.0 (Wang et al. 2010) to estimate Ks under the maximum likelihood method with “average mutation” (MA) and “most possible” (MS, model with smallest AIC_C_ score) models. The Akaike information criterion with a correction for finite sample (AIC_C_) analysis was used to select the best substitution model for alignments.

### Phylogenetic Analysis

For reconstructing the species phylogenetic tree, we randomly selected 10 genes from the 1,951 BUSCO genes common to all five genomes (see supplementary material; Gadagkar et al. 2005). Next, we aligned nucleotide sequences pairwise based on the amino acids in MUSCLE (Edgar 2004). We retained all indel mutations and concatenated all gene sequences using SequenceMatrix (Vaidya et al. 2011). In total 13,182 bp were selected.

For *Mariner-2_DF*, because some species contained no intact transposase ORF, we used BLASTn to query *Mariner-2_DF* ORFs against the corresponding genome assembly and extracted all hits ≥847 bp (80% of the length of the transposase ORF) instead. For accelerating the calculations, we removed identical copies with SDDC (Ibrahim et al. 2017). We then aligned nucleotides with MUSCLE (Edgar 2004) and retained all indel mutations.

We used both maximum likelihood (GTRGAMMA model) and Bayesian inference (GTR+I + G model) methods with 1,000 Bootstrap replicates to reconstruct the unrooted phylogeny in RAxML v8.2.12 (Stamatakis 2014) and MrBayes v3.2.6 (Ronquist et al. 2012), respectively. We used iTOL v4 (https://itol.embl.de/; last accessed September 2, 2018) for visualizing the phylogenetic trees.

### Estimation of the Neutral Mutation Rate and TE Insertion Time

To estimate the *Mariner-2_DF* proliferation time, we used the formula *T* = *k*/2*r* (Li 1997), where *T* corresponds to the insertion time in millions of years, *k* corresponds to the number of nucleotide substitutions per site, and *r* corresponds to the neutral mutation rate. If we accept that the rate of neutral evolution of TEs within a genome matches that of their host, the rate of neutral evolution for their host nuclear genes can be employed. Because the neutral mutation rate varies in different species lineages, we first estimated neutral mutation rates using species divergence times combined with the average Ks estimated from the 1,951 BUSCO genes under the MA and MS models. Divergences times were from the TimeTree of Life (TTOL) (Hedges et al. 2015): Diptera and Hymenoptera, 325 million years ago (Mya); *D. grimshawi* and *D. ficusphila*, 50 Mya; *M. rotundata* and ants, 162 Mya; and *S. invicta* and *A. echinatior* (two ants), 91 Mya. For Ks estimation in *Mariner-2_DF*, we compared the same genome copies as above with their species-specific consensus (i.e., putative ancestral) sequences (Zhang et al. 2014). Finally, using the species-specific estimated neutral mutation rates and Ks values of *Mariner-2_DF* we calculated the TE insertion time.

## Results

### Sequencing and Assembly of Fire Ant Germline Transcriptomes

Active TEs are presumably expressed in germ cells or during early development prior to germ cell specification to ensure their long-term survival (Levin and Moran 2011; Haig 2016). To identify such active TEs, we sequenced the RNA isolated from two ovaries and one testis samples, obtaining 5.0 Gb (monogyne ovaries, MO), 4.8 Gb (polygyne ovaries, PO), and 5.1 Gb (monogyne testes, MT) of raw sequences (GenBank SRA accession: SRP136925). We assembled each transcriptome separately using Trinity (Grabherr et al. 2011) and obtained 75.8 Mb (MO), 64.4 Mb (PO), and 73.1 Mb (MT) of assembled sequences. This corresponded to 31,856 (44,653 isoforms, MO), 27,487 (37,949 isoforms, PO), and 30,026 (42,447 isoforms, MT) transcripts. The three independent transcriptomes have similar assembly qualities and are probably representative of the overall germline gene expression landscape (supplementary material). These and additional statistics are summarized in supplementary table S2, Supplementary Material online.

### Identification of Highly Expressed TEs in the Fire Ant Germline

**Fig. 1.**
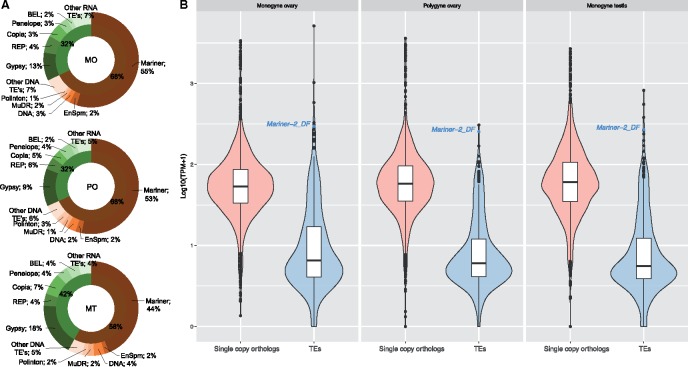
—Gene expression distributions for the fire ant germline RNA-seq data sets. (*A*) The number of putative DNA TE transcripts was more than that for retrotransposons. The *mariner* elements were the dominant TE in all samples (53% of TE transcripts in MO, 55% in PO, and 44% in MT). Pie charts shaded in green and brown are retrotransposons and DNA transposons, respectively. (*B*) Box and violin plots of log_10_(TPM + 1) values: the top and bottom of the box are the first and third quartiles (Q), respectively; the horizontal bar within the box is median. Most (>84%) of the TE-containing transcripts were expressed lower than the first Q of single copy BUSCO genes (first Q TPM: MO = 32.48, PO = 34.41, MT = 33.8) in all samples. Only 27 TE-containing transcripts in MO, 8 in PO, and 13 in MT had higher expressions level than the third Q of BUSCO genes (third Q TPM: MO = 85.8, PO = 94.6, MT = 105.5). See also supplementary table S3, Supplementary Material online.

To identify assembled transcripts containing potential TE sequence, we used RepeatMasker to compare each germline transcriptome against the Repbase repeat database (v20.09) (Bao et al. 2015). This approach permits the identification of putative TEs that are similar (≤20% nucleotide divergence) to those in Repbase but have not yet been annotated for the fire ant. After filtering potential false positive results (short matches with masked length <200 bp, divergence >20%), we obtained 565 (MO), 409 (PO), and 579 (MT) candidate TE transcripts (supplementary table S3, Supplementary Material online), corresponding to 1.77% (565/31,856; MO), 1.49% (409/27,487; PO), 1.93% (579/30,026; MT) of the total transcripts. Forty-two percent of the candidate TE transcripts were entirely or mostly (≥90%) composed of TE sequence (supplementary fig. S1, Supplementary Material online), and thus, are likely truly expressed TEs. Of the remaining 58%, those transcripts containing a low percentage of TE sequence (e.g., <60%) likely represent chimeras or misassemblies, whereas some of the transcripts with an intermediate percentage of TE sequence (60% to 90%) may be real TEs (e.g., *Mariner-2_DF* matched at 44% [MO], 64% [MT], and 78% [PO], respectively, the *Mariner-2_DF* in MO was a chimera, supplementary fig. S2, Supplementary Material online). In all three data sets, the number of putative DNA TE transcripts was more than that for retrotransposons (fig. 1*A*). The *IS630-Tc1-mariner* elements were the dominant TE in all samples (53% of TE transcripts in MO, 55% in PO, and 44% in MT).

We next assessed the approximate relative expression levels of these TE-containing transcripts by comparison to the arthropod subset of the BUSCO (Benchmarking Universal Single-Copy Orthologs) genes. We chose the BUSCO genes because they span a range of expression levels, and thus would be a suitable reference or control gene set. Comparisons within each sample revealed that >84% of the TE-containing transcripts (MO, 84.8% [479/565]; PO, 88.5% [362/409]; MT, 87.9% [509/579]) were expressed at levels below the 25th percentile of the BUSCO genes (“lowly expressed,” 25th percentile TPM: MO = 32.5; PO = 34.4; MT = 33.8). Very few TE-containing transcripts were expressed above the 75th percentile of BUSCO genes (“highly expressed,” 75th percentile TPM: MO = 85.8; PO = 94.6; MT = 105.5): 27 (MO), 8 (PO), and 13 (MT) transcripts (fig. 1*B*, supplementary table S3, Supplementary Material online). These corresponded to 32 nonredundant transcripts. Here, it should be noted that “highly expressed” is at the TE “gene” level whereas per element expression could be lower. We excluded 20 TE candidates from further analysis because they lacked a detectable transposase or RT-ORF and are thus nonautonomous; we wanted to focus on autonomous TEs (supplementary table S4, Supplementary Material online). The remaining 12 contained transposase ORFs with 11 having complete TE structures (ORF plus terminal repeats) in the fire ant genome (table 1). Ten of these eleven TEs were DNA TEs, and of these, all were in the *IS630-Tc1-mariner* superfamily. Interestingly, only one TE, *Mariner-2_DF*, was highly expressed relative to the BUSCO genes in all three independent samples (table 1).

**Table 1:** Highly Expressed Autonomous TEs in *S. invicta*

TE	Class	TE Length (bp)	TPM (Transcriptome)			Copy Number in *S. invicta* Genome (Coverage > 60%)	Genetic Diversity (π) in *S. invicta* Genome	Identical Copies within Genome	Top 20 TE (Repbase)
			MO	PO	MT				
*Mariner-2_DF*	DNA	1,322	**295.43**	**253.47**	**267.88**	857	0.0051 ± 0.0002	30	•
*Mariner-9_SIn*^a^		1,293	**194.01**	67.95	**820.16**	16	0.1003 ± 0.0042	0	
*Mariner-12_SIn*^a^		1,284	**103.69**	63.70	88.44	110	0.05 ± 0.0015	0	•
*Mariner-30_Sin*		1,286	**94.62**	81.25	71.96	306	0.026 ± 0.0007	2	•
*Mariner-35_Sin*		2,080	23.50	**168.68**	**263.36**	8	0.0737 ± 0.0028	0	
*Mariner-36_SIn*^b^		3,851	**91.59**	65.05	4.03	2	0.0309 ± 0.0033	0	
*Mariner-37_Sin*		1,321	**237.07**	42.05	52.58	227	0.0304 ± 0.008	0	•
*Mariner-4_AEc*		1,287	**160.85**	67.46	91.69	284	0.0427 ± 0.0013	1	•
*Mariner-14_HSal*^b^		1,271	**193.09**	**104.62**	68.87	5	0.1168 ± 0.0069	0	•
*Mariner-50_HSal*		1,313	81.12	69.47	**379.50**	22	0.0082 ± 0.0009	0	
*Gypsy-13_SI-I*^a^	RNA	4,661	**113.22**	93.65	58.44	5	0.0081 ± 0.0015	0	

Note.–“Highly-expressed” TPM values are marked in bold. All TE consensus sequences that we reconstructed are available in the supplementary information.

a Consensus sequences are the same as sequences from Repbase.

b Sequences from Repbase.

As a second approach to identifying highly expressed TEs, we aligned the three germline RNA-seq data sets directly to the Repbase v20.09 TE sequences (Bao et al. 2015). Using only TEs as the reference gene set has the advantages of avoiding chimeras and allowing direct comparisons of the TPM expression values. However, TE expression levels relative to the rest of the transcriptome cannot be assessed and only curated TEs (or those within mapping tolerance) will be detected. Of the top 20 TEs in each of the three germline data sets, 6 nonautonomous and 12 autonomous TEs were shared (supplementary table S5, Supplementary Material online). Of the latter 12, 6 were also identified in our approach one (table 1). Again, *Mariner-2_DF* was identified as the most highly expressed TE in all three data sets and much more than the next most expressed TE (>4-fold, supplementary table S5, Supplementary Material online).

### Indirect Evidence for the Recent Proliferation of *Mariner-2_DF* in the Fire Ant Genome

TE activity can be indirectly inferred from sequence divergence among the elements. Multiple TE insertions with identical sequences in the host genome would suggest that the focal TE likely was recently active. We searched for identical copies for each of the 17 (11 from approach one, and 6 additional from approach two) highly expressed autonomous TEs in the fire ant genome and found only *Mariner-2_DF*, *Mariner-30_SIn*, *Mariner-5_SIn*, and *Mariner-4_AEc* had identical TE copies (table 1). The remaining 13 TEs, despite having an intact transposase ORF, did not have additional identical copies, possibly implying that they are no longer active or have been post-transcriptionally silenced by the host genome; some high TE expression may be due to read-through from (or possibly chimerism with) a neighboring gene. *Mariner-2_DF* had 9 “variants” with multiple copies (2–11 identical copies for 30 total) in the fire ant genome, more than *Mariner-30_SIn* (two variants with two copies each), *Mariner-5_SIn* (two variants with two copies each), and *Mariner-4_AEc* (one variant with two copies).

**Fig. 2.**
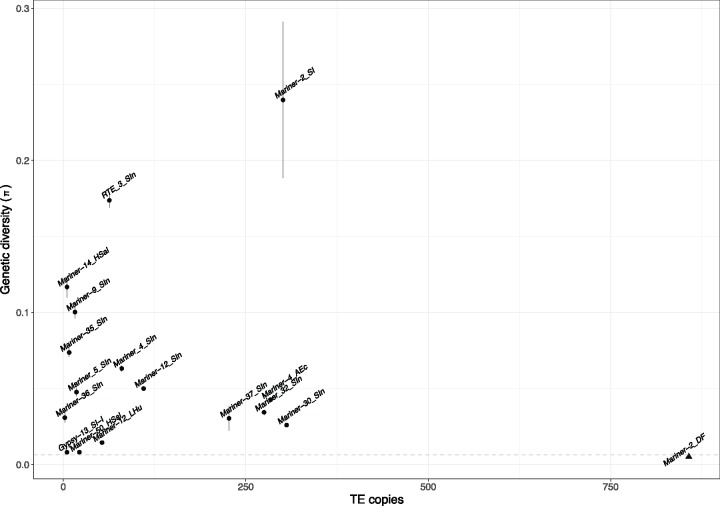
—The genetic diversity of 17 highly expressed autonomous TEs in the fire ant genome. *Mariner-2_DF* (triangle) had the lowest genetic diversity (π = 0.005  ±  0.0002) and the highest copy number (*n* = 857, coverage ≥60%) compared with other highly expressed TEs. Dots and triangle indicate estimated π; vertical lines indicate standard error.

Recently proliferating elements are predicted to have low genetic diversity (π) among TE copies and may have many copies in the genome. Examination of the mean genetic diversity of the 17 highly expressed autonomous TEs in the fire ant genome revealed that *Mariner-2_DF* had the lowest mean genetic diversity (0.005; all others 0.008 to 0.240). *Mariner-2_DF* also had the highest copies (*n* = 857; all others *n* ≤ 306; sequence ≥60% of full length). Together, these observations suggest that *Mariner-2_DF* has expanded most recently in the fire ant genome (fig. 2; table 1).

### High Polymorphism in *Mariner-2_DF* TE Insertion Sites in the Fire Ant

The above analysis indicated that *Mariner-2_DF*, and possibly *Mariner-30_SIn*, *Mariner-5_Sin*, and *Mariner-4_AEc*, may have been recently active. We next asked if any of them are currently active. Evidence supporting this would be the observation of TE insertion site polymorphisms. We searched for TE insertions in the low-coverage genome sequences from brothers of seven fire ant families from Georgia, USA (Wang et al. 2013) using ngs_te_mapper (Linheiro and Bergman 2012). Among our 17 highly expressed TE candidates, we detected only *Mariner-2_DF* with nonreference insertions (*N* = 70, supplementary fig. S3, Supplementary Material online). This finding accords well with our observation that *Mariner-2_DF* was the only highly expressed TE in all three germline transcriptomes.

**Fig. 3.**
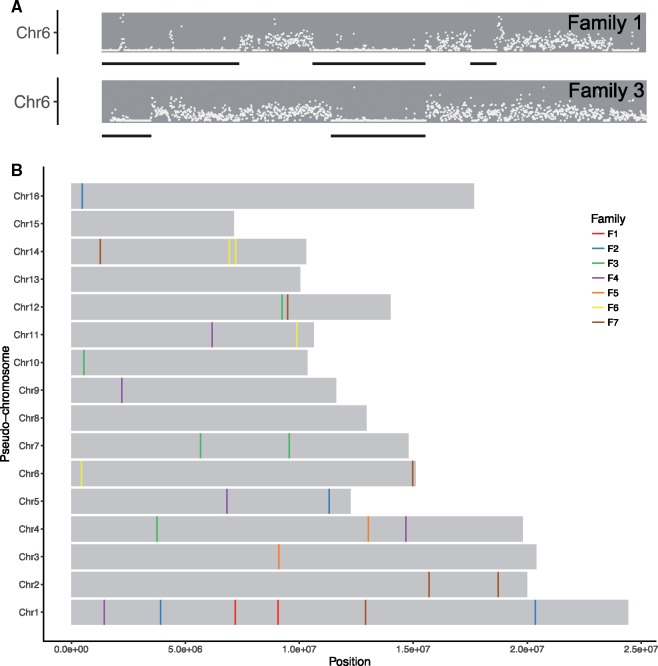
—Non-reference *Mariner-2_DF* insertions. (*A*) Identification of IBD regions. SNP density between brothers along fire ant pseudochromosomes was used to identify likely IBD regions (black underlines) based on low SNP density. Each dot represents the SNP density per 10 kb window. Pseudochromosome 6 from families 1 and 3 are shown as examples. All chromosomes and ant families are shown in supplementary figs. S4–S10, Supplementary Material online. (*B*) Summary of nonreference *Mariner-2_DF* insertions in the fire ant genome for seven fire ant families. We found 28 family-specific nonreference *Mariner-2_DF* insertions in *S. invicta* genome. There were at least two unique insertion sites in each ant family, indicating high *Mariner-2_DF* insertion polymorphism in fire ants. See also supplementary figs. S4–S10, supplementary table S6, Supplementary Material online.

TE insertion polymorphism may also be due to an “old” insertion that is still segregating in the population. Thus, we next categorized nonreference TE insertions into those found only in one or both brothers of an ant family (“family-specific”) or in ≥2 families (“common”). We found that 42 insertions (60%) were common, and for these we cannot exclude that they were due to segregation of common alleles. Stringent filtering revealed 28 (40%) robust nonreference *Mariner-2_DF* single-family insertions in our data set (fig. 3*B*, see also supplementary figs. S4–S10, supplementary table S6, Supplementary Material online). Importantly, we found that every family had unique insertions (supplementary figs. S4–S10), indicating high *Mariner-2_DF* polymorphism rates, and hence, indirect evidence for current transposition.

Finally, we looked for completely de novo insertions occurring in the germline of the mother. This can be identified by examining IBD regions (see Materials and Methods and fig. 3*A*). We did not find any, indicating that the *Mariner-2_DF* insertion rate is <∼1/9 genomes per generation (number of individuals × average fraction of genome in IBD per brother pair = 14 × 0.62). Although we did not find evidence for a new insertion, we did find evidence for a somatic excision; for one locus in individual F7_b both reads with and without *Mariner-2_DF* were observed (supplementary fig. S11, Supplementary Material online).

Our combined results so far indicated that *Mariner-2_DF* was probably the most active DNA TE in the fire ant genome, and thus we focused the remainder of our analyses on *Mariner-2_DF*.

### Patchy Distribution of *Mariner-2_DF* in Insects

Based on the above analyses, we found that *Mariner-2_DF* is a TE having a complete transposase and is highly expressed in all three germlines. The fire ant *Mariner-2_DF* homolog is extremely similar to the original *Mariner-2_DF* (99.09%) reported in *Drosophila ficusphila* (Bao et al. 2015). This suggested a recent HTT event. Thus, we examined the distribution of *Mariner-2_DF* in insects. We surveyed 52 arthropod genome assemblies and detected *Mariner-2_DF*-like sequences with hit length >100 bp and identity >90% in eight species across three insect orders (Diptera, Hymenoptera, and Hemiptera). The eight species included four *Drosophila*: *D. yakuba*, *D. erecta*, *D. ficusphila*, and *D. grimshawi*; three Hymenoptera: *Megachile rotundata*, *Acromyrmex echinatior*, and *S. invicta*; and one Hemiptera, *Rhodnius prolixus* (BLASTn identity: 90–99%, table 2). We could not detect *Mariner-2_DF*-like sequences in the other 44 arthropods (Diptera, *n* = 25 including 20 *Drosophila*; Lepidoptera, *n* = 2; Coleoptera, *n* = 1; Hymenoptera, *n* = 12; Hemiptera, *n* = 1; Phthiraptera, *n* = 1; Ixodida, *n* = 2). Thus, *Mariner-2_DF* clearly has a patchy distribution in insects consistent with HTT events.

**Table 2:** The Identification of *Mariner-2_DF*-Like Sequences in Insect Genome Assemblies

Order	Family	Species	Common Name	Biogeographic Realm	Native Distribution	Nucleotide Identity of the Best Hit against *Mariner-2_DF* Consensus from Repbase	Copy Numbers (Coverage ≥ 40%)
Diptera	Drosophilidae	*Drosophila yakuba*	Fruit fly	Afrotropic	Africa	273/305 (90%)	0
		*Drosophila erecta*		Afrotropic	West Africa	352/387 (91%)	0
		*Drosophila ficusphila*		Palearctic	East Asia, Australian	1,240/1,303 (95%)	69
		*Drosophila grimshawi*		Oceania	Hawaii	1,306/1,322 (99%)	82
Hymenoptera	Megachilidae	*Megachile rotundata*	Alfalfa leafcutter bee	Palearctic	Europe and Asia	1,217/1,325 (92%)	2
	Formicidae	*Acromyrmex echinatior*	Panamanian leafcutter ant	Neotropic	Central America	1,250/1,324 (94%)	58
		*Solenopsis invicta*	Red imported fire ant	Neotropic	South America	1,311/1,323 (99%)	980
Hemiptera	Reduviidae	*Rhodnius prolixus*	Kissing bug	Neotropic	Central and South America	524/573 (91%)	26

### Characterization of *Mariner-2_DF* in Each Species

In order to identify the structural features of the *Mariner-2_DF* sequence in the eight species, we reconstructed the species-specific consensus sequences from all *Mariner-2_DF* sequence fragments ≥100 bp in each genome. For *D. yakuba*, *D. erecta*, and *R. prolixus*, we could only detect remnants of the *Mariner-2_DF* sequence.

In the remaining five species (*D. ficusphila*, *D. grimshawi*, *M. rotundata*, *A. echinatior*, and *S. invicta*), we were able to generate full-length *Mariner-2_DF* consensus sequences. Pairwise comparisons of the consensus sequences amongst the species revealed high identity (91.63–99.09% nucleotide identity; 89.52–99.43% amino acid identity; supplementary table S7, Supplementary Material online). All consensus sequences have an intact open reading frame including the characteristic signatures of *mariner* family transposons: two conserved helix-turn-helix (HTH) DNA binding motifs, the catalytic domain harboring a DD34D motif, and a C-terminal YSPDLAP amino acid motif (Plasterk et al. 1999) (supplementary fig. S12, Supplementary Material online), as well as highly similar TIRs (>93%, supplementary fig. S13, Supplementary Material online). The high sequence conservation of the transposase domains and TIRs suggest that all these fragments were derived from the same element via horizontal transfer. Horizontal transfer of *Mariner-2_DF*, which corresponds to *Dromar8Mfic*, has previously been reported within *Drosophila* species (*Dromar8*) and *R. prolixus* (*Rpmar57*) (et al.Filée et al. 2015; Wallau et al. 2014, 2016).

### No Evidence for Purifying Selection of *Mariner-2_DF* within the Host Genomes

Although high nucleotide identity of *Mariner-2_DF* within each of the five species could be the result of recent transposon expansion, another remote alternative explanation could be extremely strong purifying selection to maintain the same sequence. We tested for evidence of strong purifying selection using the codon-based *Z*-test and found no support (all *P *>* *0.05, FDR adjusted). Therefore, the high identity of *Mariner-2_DF* is most simply explained by recent proliferation within each genome.

### Nucleotide Divergence at Synonymous Sites

To provide additional evidence of horizontal transfer of *Mariner-2_DF*, we conducted interspecies Ks comparisons of *Mariner-2_DF* and 1,951 orthologous nuclear genes (BUSCO genes) common to all five insect species (supplementary table S8, Supplementary Material online). Synonymous substitutions are generally considered nearly neutral and accumulate with divergence time. All genes in a genome should have similar rates of synonymous substitutions (Ks) when compared between species because the genomes diverged at the same time. This would also be the case for a TE that is already present in the genome. However, a TE arriving by horizontal transfer would be expected to have fewer synonymous substitutions than the other genes in the same genome.

**Fig. 4.**
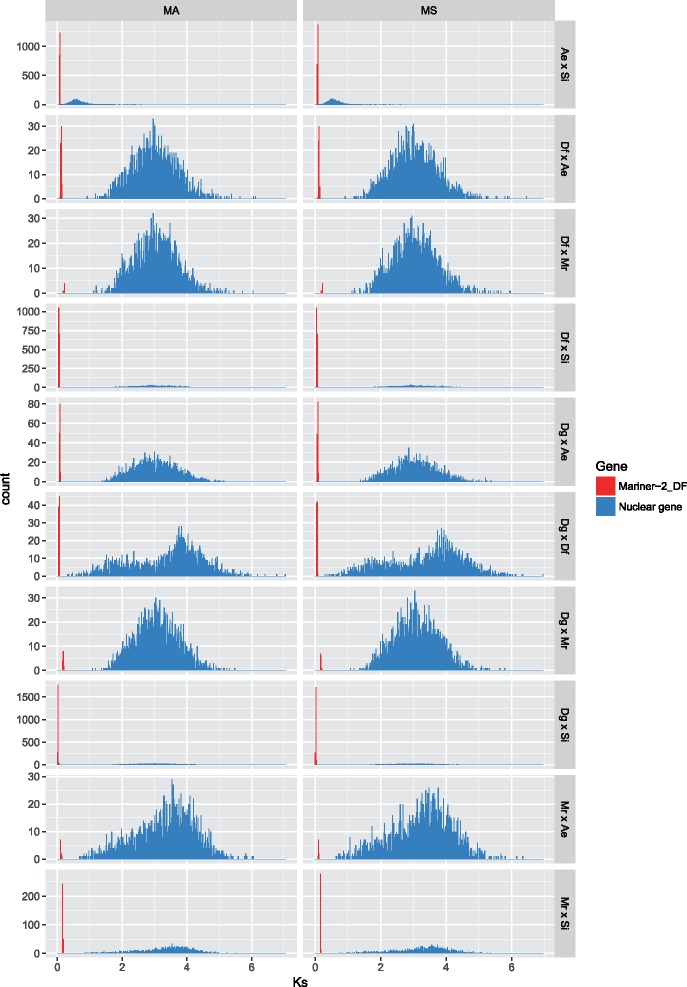
—Histogram of pairwise comparisons of the synonymous substitution rate (Ks) in five *Mariner-2_DF* containing insects. *Mariner-2_DF* has accumulated significantly fewer synonymous mutations in all comparisons (Games-Howell post-hoc test, all *P* value < 10^−4^) suggesting multiple horizontal events. MA: average mutation model for Ks estimation. MS: uses the smallest AIC_C_ mutation model for Ks estimation. Ae: *Acromyrmex echinatior*, Si: *Solenopsis invicta*, Df: *Drosophila ficusphila*, Dg: *D. grimshawi*, Mr: *Megachile rotundata*. Nuclear gene: 1951 shared single copy BUSCO genes. Number of *Mariner-2_DF* pairs: Ae × Si = 2,810; Df × Ae = 60; Df × Mr = 6; Df × Si = 1,686; Dg × Ae = 140; Dg × Df = 84; Dg × Mr = 14; Dg × Si = 3,934; Mr × Ae = 10; Mr × Si = 281. *Y*-axis scale is different among species pairs.

We used two different Ks estimation methods, the “average mutation” model (MA) and the “most possible” model (MS, model has the smallest AIC_C_ score), to assess nucleotide divergence. In the MA model, the software averages the Ks from 203 time-reversible models, thereby reducing biases arising from model selection. In the MS model, Ks is estimated from the best model based on the Akaike information criterion with a correction for finite sample size (AIC_C_). In our analysis, both methods showed the same result: inter-species Ks values were significantly lower for *Mariner-2_DF* than for nuclear genes in all species-pair comparisons (Games-Howell post-hoc test, all *P* value < 10^−4^, fig. 4). Thus, our results strongly support multiple cases of horizontal transfer for *Mariner-2_DF*.

### Phylogenetic Incongruence

**Fig. 5.**
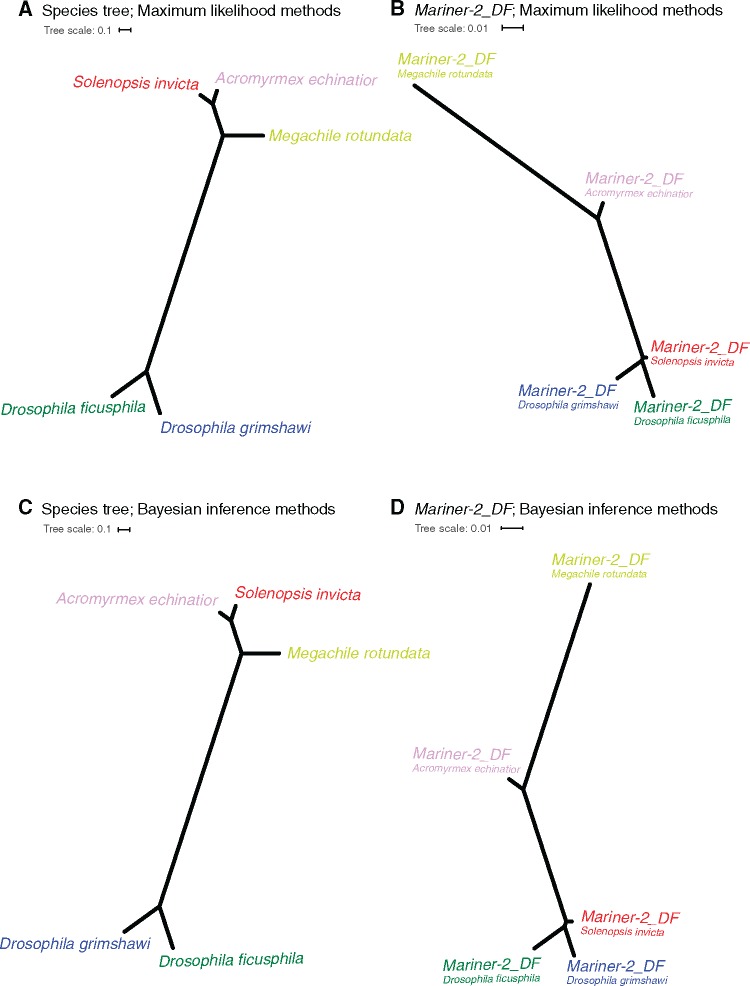
—Phylogenetic incongruence between the species tree and the *Mariner-2_DF* transposon tree. The species tree was based on the concatenated nucleotide sequences of 10 BUSCO genes (*A*, *C*), and the *Mariner-2_DF* tree was based on the transposase sequences (*B*, *D*; all copies with length ≥847 bp). The unrooted phylogenetic tree was constructed by the Maximum-likelihood (*A*, *B*) and Bayesian inference methods (*C*, *D*) under the GTR+GAMMA and GTR+I + G models, respectively. The five species are color coded: *A. echinatior* (pink), *D. ficusphila* (green), *D. grimshawi* (blue), *S. invicta* (red), and *M. rotundata* (light-green).

Discordance between the species tree with the TE gene tree would be another line of evidence for HTT. We used both maximum likelihood and Bayesian inference to construct species trees for the five focal species based on the concatenated sequences for 10 randomly selected BUSCO genes, a subsampling method that is relatively robust for these clearly separated species (Gadagkar et al. 2005). Trees generated by both methods matched the well-established phylogeny for these five species, placing the two ants (*S. invicta* and *A. echinatior*) closest to each other (fig. 5). In contrast, the phylogenetic trees for the *Mariner-2_DF* sequences presented an incongruent topology, placing *S. invicta* closer to the *Drosophila* species rather than the other ant, *A. echinatior*. Together these results support evolutionarily recent HTT for *Mariner-2_DF*.

### Invasion Dates of *Mariner-2_DF*

**Fig. 6.**
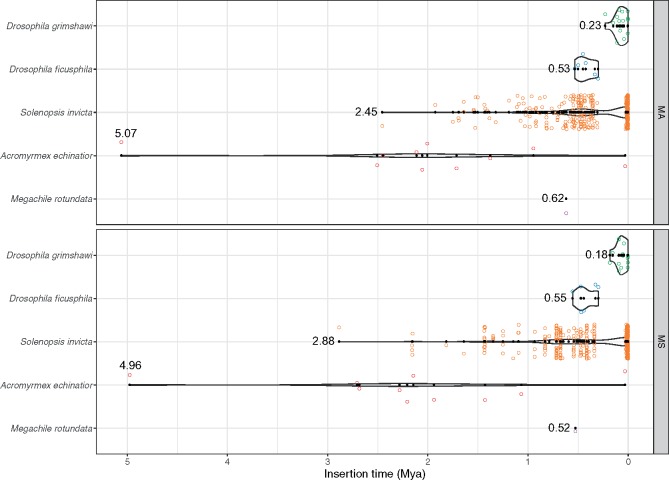
—Estimates of *Mariner-2_DF* insertion times and proliferation profile. Time of *Mariner-2_DF* insertion in each species was estimated with the formula *T* = *k*/2*r* (Li, 1997). We estimated mutation rate (*r*) using 1,951 nuclear genes in each species. The estimated horizontal insertion times are indicated. Mya: million years ago. Individual *Mariner-2_DF* insertions are shown as open circles and jittered. Age of *Mariner-2_DF* genome copies are shown with black dots. MA: average mutation model for Ks estimation. MS: the smallest AIC_C_ mutation model for Ks estimation.

To estimate the date of *Mariner-2_DF* invasion into each of the five host genomes, we first calculated species-specific neutral mutation rates (*r*) from the average Ks estimates from 1,951 BUSCO genes (above) and species divergence times from the TTOL (Hedges et al. 2015). The substitution rates of each species were 5.17 × 10^−9^ (MA) and 5.22 × 10^−9^ (MS) in *Drosophila*, and 1.02 × 10^−8^ (MA) and 1.00 × 10^−8^ (MS) in bees, 3.53 × 10^−9^ (MA) and 3.27 × 10^−9^ (MS) in ants (supplementary table S9, Supplementary Material online). Then, using these substitution rates as *r* in the formula *T* = *k*/2*r* (Li 1997), we estimated that *Mariner-2_DF* entered into *D. grimshawi* about 0.23 (MA) − 0.18 (MS) Mya (million years ago), *D. ficusphila* 0.55 (MS) − 0.53 (MA) Mya, *M. rotundata* 0.62 (MA) − 0.52 (MS) Mya, *A. echinatior* 5.07 (MA) − 4.96 (MS) Mya, and *S. invicta* 2.88 (MS) − 2.45 (MA) Mya (fig. 6). Interestingly, in *S. invicta*, *Mariner-2_DF* stopped jumping at ∼0.3 Mya but re-proliferated again at ∼0.026 Mya (fig. 6), and these very recent proliferations account for 47.3% (157/332) of the intact *Mariner-2_DF* copies in *S. invicta*.

## Discussion

We report an analysis of the TE content of both the ovary and testis germline transcriptomes for the fire ant, which is the first for a hymenopteran insect. A previous study profiled only ovary gene expression in honeybees (Niu et al. 2014) and did not examine TEs. Additionally, our study is one of a few insect germline transcriptomes outside of *Drosophila* and mosquitoes (Akbari et al. 2013; Yang and Xi 2017). We also report the discovery of a rare case of a currently active TE after a recent HTT (<3 My) in insects. This adds to the few cases of HTT documented for Hymenoptera (Dotto et al. 2015, 2018). Our study shows that profiling germline expression may be a potential approach for identifying active TEs.

Our analysis revealed that ∼50% of TE-containing transcripts in both the female and male germlines of the fire ant contained sequence from members of the *IS630-Tc1-Mariner* superfamily (fig. 1*A*). Although previous studies suggested that *mariners* were typically inactivated in eukaryote genomes (Feschotte and Pritham 2007; Muñoz-López and García-Pérez 2010; Yang and Xi 2017), our results are consistent with the fact that all six known cases of active *mariners* in animals are from invertebrates (Muñoz-López and García-Pérez 2010). Our findings also corroborate the previous observation that the *mariner* family is widespread in insects (Robertson 1993; Peccoud et al. 2017).

Active TEs are a genomic burden, and consequently, organisms have evolved defense mechanisms against TEs (Levin and Moran 2011; Yang and Xi 2017). Consistent with control by host defenses, >84% of the TE containing transcripts in our study were expressed at low levels (fig. 1*B*). Self-regulation could also be occurring (Kidwell and Lisch 2001; Bire et al. 2016). Nevertheless, 17 autonomous TEs may have escaped, or are not yet subject to, host defenses as they are highly expressed in the germline.

Of these, we found *Mariner-2_DF* particularly interesting because it may still be active in *S. invicta*, and possibly in a recent phase of expansion. Six lines of evidence strongly support this possibility. First, it has high germline expression and is the only one expressed in all three germline samples based on comparison to the BUSCO genes; and it has the highest germline expression in all three samples using Repbase as the reference (supplementary table S5, Supplementary Material online). Second, of the 17 highly expressed TEs examined, it is the only one with nonreference copies. Third, it has multiple unique insertion polymorphisms in seven fire ant families (fig. 3*B*, supplementary figs. S4–S10, Supplementary Material online). We found at least two insertions per family, which is likely an underestimate because our analysis only surveyed the ∼67% of the scaffolds joined into pseudochromosomes. Fourth, it can undergo somatic excision (supplementary fig. S11, Supplementary Material online). Fifth, it is the TE with the most copies (*n* = 857; all others *n* ≤ 306; sequences ≥60% of full length). This copy number is similar to other *mariner* lineages in *Drosophila* (e.g., ∼460 copies of *Dromar6* in *D. erecta*) that are likely in a recent phase of expansion (Wallau et al. 2014). Finally, it has the lowest intercopy genetic diversity, including many identical copies, in the fire ant genome (fig. 2, table 1). The low genetic diversity among the *Mariner-2_DF* copies suggests that it may be the youngest active *mariner* in fire ant genome. This also indicates that the fire ant has not yet evolved a strong defense against *Mariner-2_DF*.

Although we were successful in discovering one active TE, our analysis may have underestimated the number of active TEs in fire ants for several reasons. For example, we selected for highly expressed TEs in our analysis, thus we would miss active but moderately or lowly expressed TEs. Related, we only profiled one time point for the ovaries (virgin adults) and testes (third and fourth instar), so TEs expressed at other developmental times or during periods of stress (e.g., Naito et al. 2009) would also be missed. Likewise, we did not examine testes from the *Sb* genotype. Additionally, although we used an improved fire ant genome, there are still assembly gaps, precisely where TEs are typically overrepresented. TE polymorphism (an indication of activity) in the gaps would be undetected. Similarly, fire ant centromeres occupy a third of the genome (Huang et al. 2018), and any polymorphic insertions there would be difficult to detect.

In addition to contemporary *Mariner-2_DF* activity in the fire ant, this transposon may have been horizontally transferred into several other species recently (<5.1 My). With the caveat that the analyzed genome assembly qualities were variable, thereby possibly introducing false negatives in *Mariner-2_DF* presence and sequence completeness, our investigation of its taxonomic distribution revealed a patchy distribution, being found in eight species among 52 diverse insects. For three of the eight species, only remnants of the *Mariner-2_DF* transposon sequence were detected, indicating host inactivation of the transposon and possibly suggesting an older horizontal transfer date. For the remaining five species, there was high sequence identity among the species and fewer synonymous substitutions in *Mariner-2_DF* than in nuclear genes in pairwise comparisons, suggesting at least five independent relatively recent horizontal transfer events (fig. 4). Intact full-length *Mariner-2_DF* sequences were only detected in *S. invicta* and *D. grimshawi* (the youngest, ∼0.18–0.23 My), suggesting that *Mariner-2_DF* may potentially be active in only these two species. Our results match previous studies reporting HTT for *Mariner-2_DF* in *D. ficusphila* (*Dromar8Mfic*), *D. grimshawi* (*Dromar8*) (Wallau et al. 2014, 2016) and *R. prolixus* (*Rpmar57*) (Filée et al. 2015).

HTT is a well-documented phenomenon among insects. A recent study found that some insects have large proportions of the genome from HTT (24.69% in the stable fly, *Stomoxys calcitrans*), but in fire ants this value is only 0.75% (Peccoud et al. 2017). In general, previous research proposed that closely interacting species are more likely to exchange TEs (Soucy et al. 2015). HTT seems unlikely to have occurred directly among the eight species examined in our study because they have no documented direct ecological interactions. Nevertheless, the current native geographic ranges for *R. prolixus* and the two ants may overlap (table 2) and historical geographic ranges may have overlapped for the other species, possibly permitting HTT. More likely, HTT occurred indirectly through one or a series of common vectors between recipient species. These could include viruses, such as baculoviruses or the flock house virus, which are known to carry TEs (Loreto et al. 2008; Routh et al. 2012; Gilbert et al. 2014), and intimately associated parasites, *Wolbachia*, or other TEs (Houck et al. 1991; Loreto et al. 2008; Schaack et al. 2010; Venner et al. 2017). We did check a phoretic mite of fire ants, *Histiostoma blomquisti* (Sokolov et al. 2003; Wirth and Moser 2010), which is commonly attached between or under the abdominal tergites of queens. However, we can exclude this mite as the vector because genome sequencing revealed no *Mariner-2_DF* copies (Lee and Wang 2016 and unpublished genome).

The direction of HTT, either direct or indirect, among the eight species examined is not clear from our study. Nevertheless, one possibility is that the three species (*D. yakuba, D. erecta*, and *R. prolixus*) containing only highly fragmented, and presumably fairly old, copies of *Mariner-2_DF*, could have been the source for the HTT events into the other five species. Related, and compatible with the first possibility, is that the two ants, which have estimated *Mariner-2_DF* colonization dates of >2.6 Mya, could have been the source for the three species with more recent insertion dates (*D. ficusphila, D. grimshawi*, and *M. rotunda*; all <0.57 Mya). Future studies incorporating additional genomes are needed to resolve this issue.

Periods of active transposition may disproportionately shape the host’s genome, leading to increased host genome diversity. Associations between bursts of TE activity and species radiations has been proposed in apes, rodents, and bats (Warren et al. 2015). Given the evolutionary recent proliferation of *Mariner-2_DF* and the high likelihood that it is currently active, highly expressed, and highly polymorphic, we suggest that, of all the TEs, *Mariner-2_DF* has been disproportionately affecting the fire ant genome. An intriguing question would be: Has this transposon generated beneficial mutations in the fire ant genome that have contributed to its adaptation to the invasive ranges? This topic will be the subject of future experiments and analyses.

## Supplementary Material

Supplementary data are available at *Genome Biology and Evolution* online.

## Supplementary Material

Supplementary Data
