# Supplementary material for: Rapid Expansion of a Highly Germline-Expressed Mariner Element Acquired by Horizontal Transfer in the Fire Ant Genome
Source: Genome Biol Evol. 2018 Oct 9;10(12):3262–78. doi: 10.1093/gbe/evy220 (PMC6307670; doi:10.1093/gbe/evy220)
Supplement: Supplementary Data [file evy220_supp.zip › Supplementary_information_HTT_20180902_2.docx]

**­­­Supplementary information**

**Rapid expansion of a highly germline-expressed *mariner* element acquired by horizontal transfer in the fire ant genome**

Chih-Chi Lee and John Wang

**Supplementary Figures**


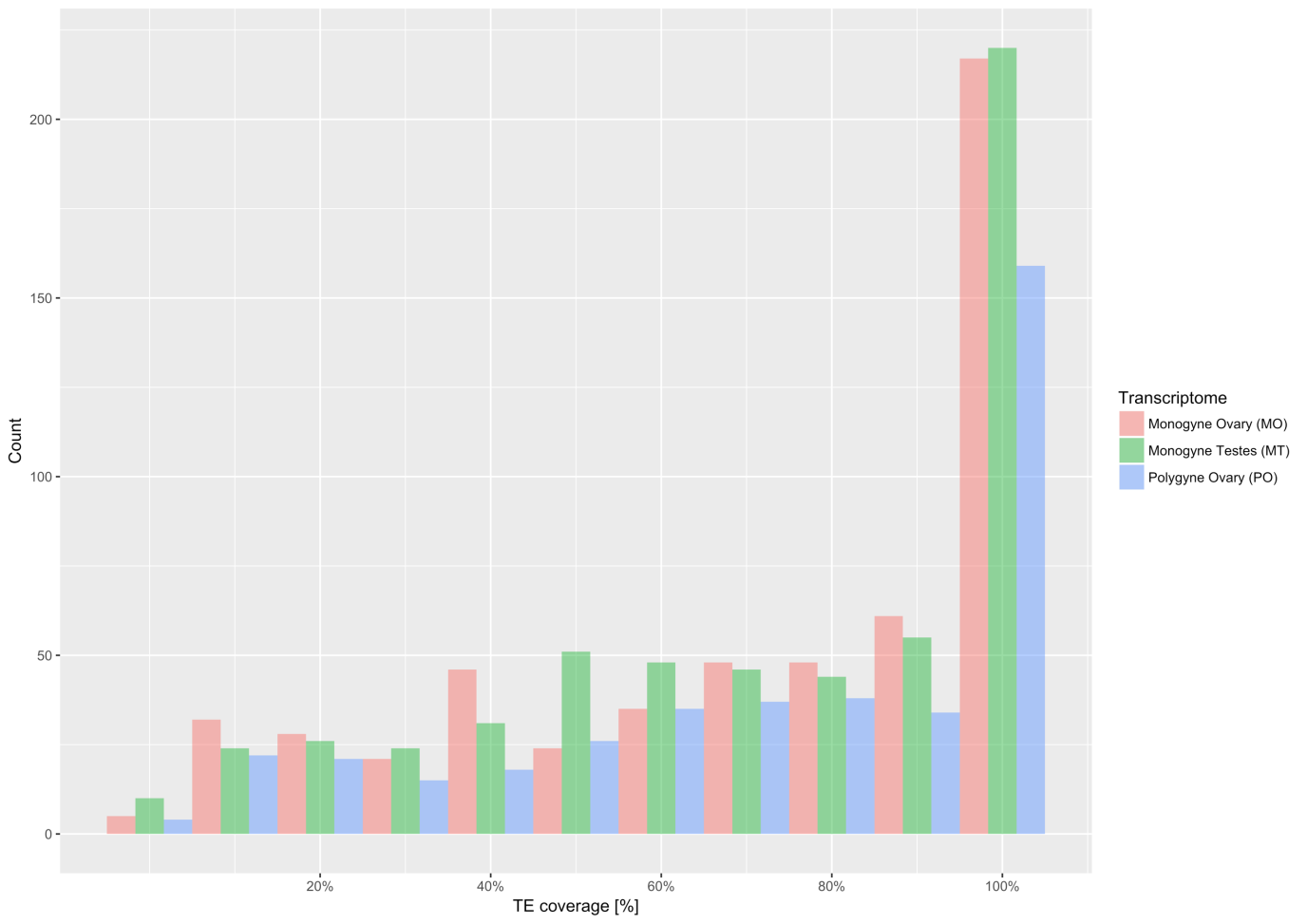


**Figure S1. TE coverage distribution in transcriptome.** Forty-two percent of the TE-containing transcripts contained over 90% TE sequence in the full length transcript.

**Figure S2. Example of a misassembly merging a TE with a gene.** Transcripts containing intermediate levels (40%-90%) of TE sequence are likely misassemblies. This case shows that the expression coverage of *Mariner-2_DF* is higher than the merged gene with only few reads linking the two, indicative of misassembly.

**Figure S3. Detection of non-reference TE insertions with low-coverage sequencing datasets.** This example illustrates a non-reference transposon insertion at position 1,451,839 nt on pseudo-chromosome 1 in family 4. The novel insertion in individual F4_B was annotated by ngs_te_mapper (A); this result was supported by BWA mapping where no reads crossed the insertion site (B). In contrast, another individual (F4_b) did not have this TE insertion, and thus no signal was detected by ngs_te_mapper (C). The BWA alignments showed DNA reads crossing the insertion site (D).

**Figure S4-S10. Detection of non-reference TE insertions with low-coverage sequencing datasets in each family.** White dots are SNP density per 10 Kb window. Color bars indicate insertion positions of non-reference *Mariner-2_DF* in Identical-by-descent (blue) or heterozygous (red) segments.


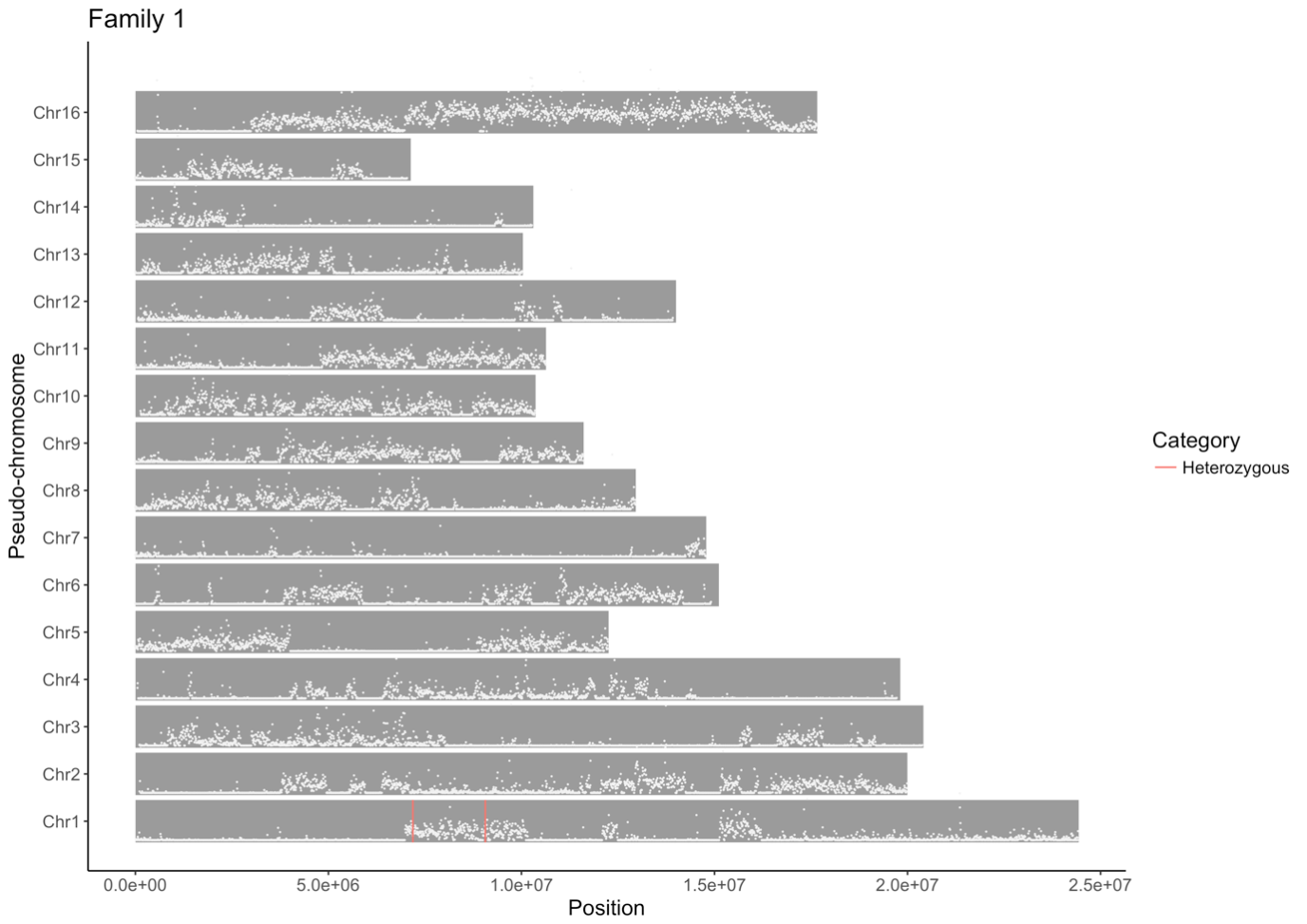


**Figure S4.**


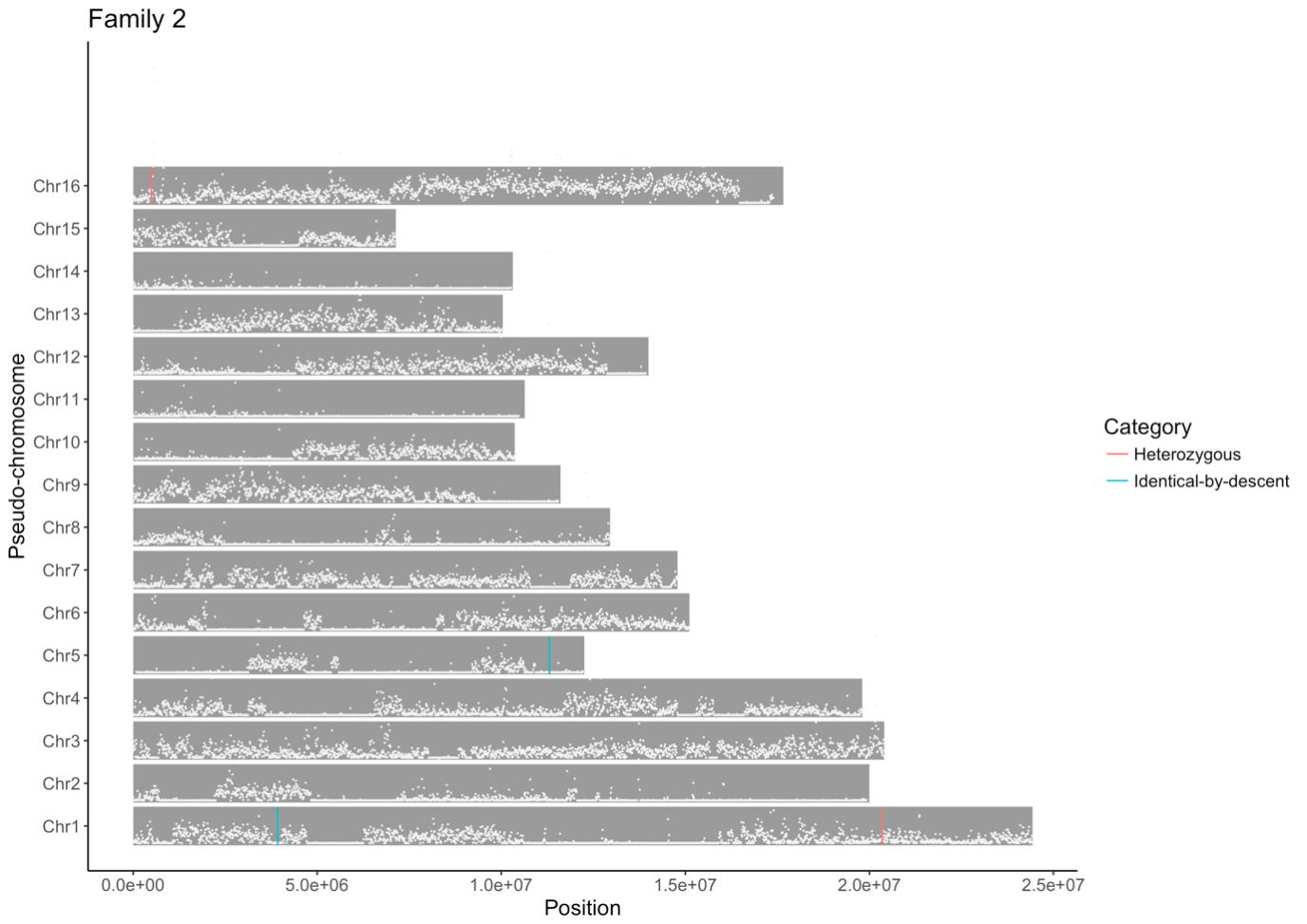


**Figure S5.**


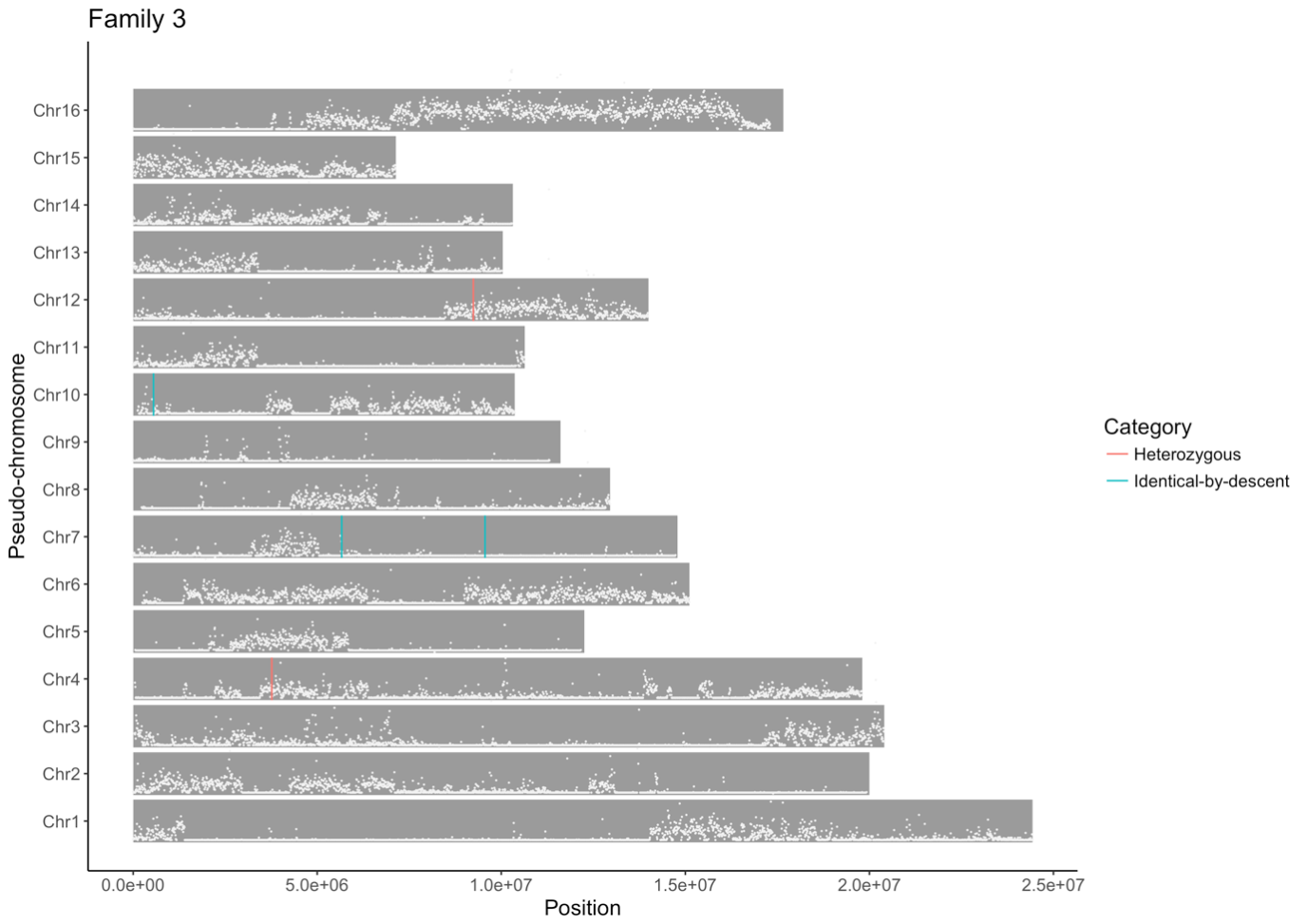


**Figure S6.**


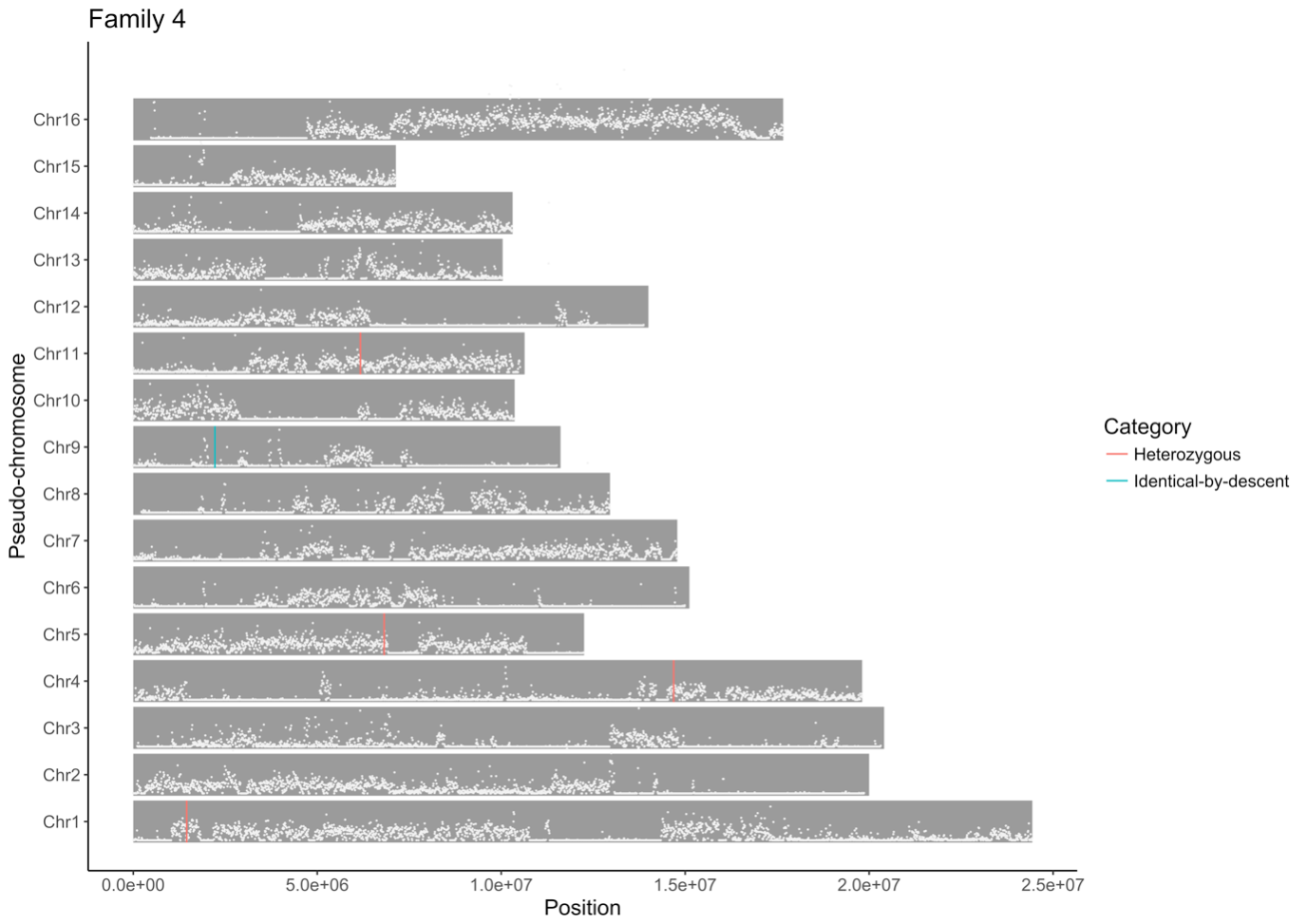


**Figure S7.**


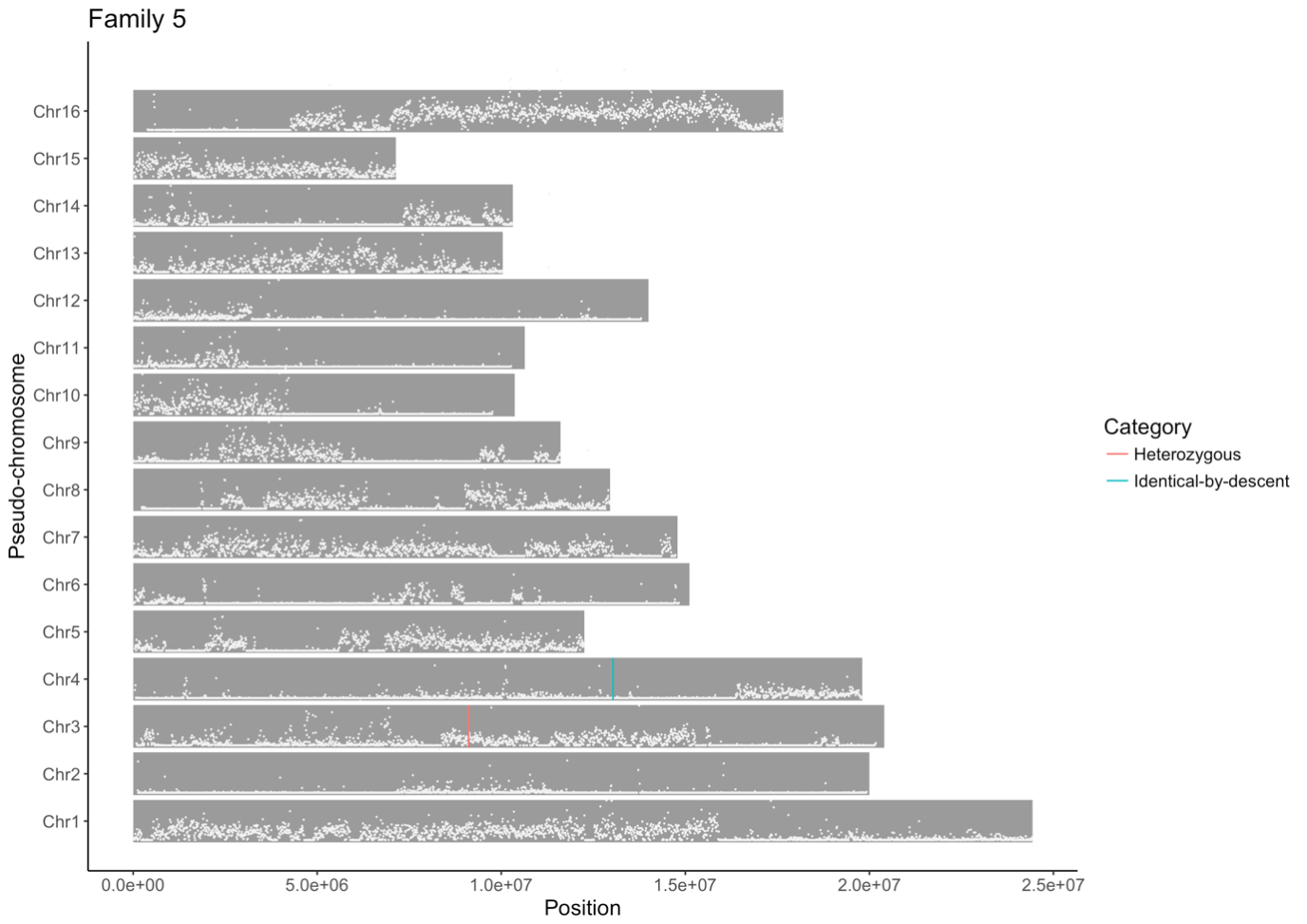


**Figure S8.**


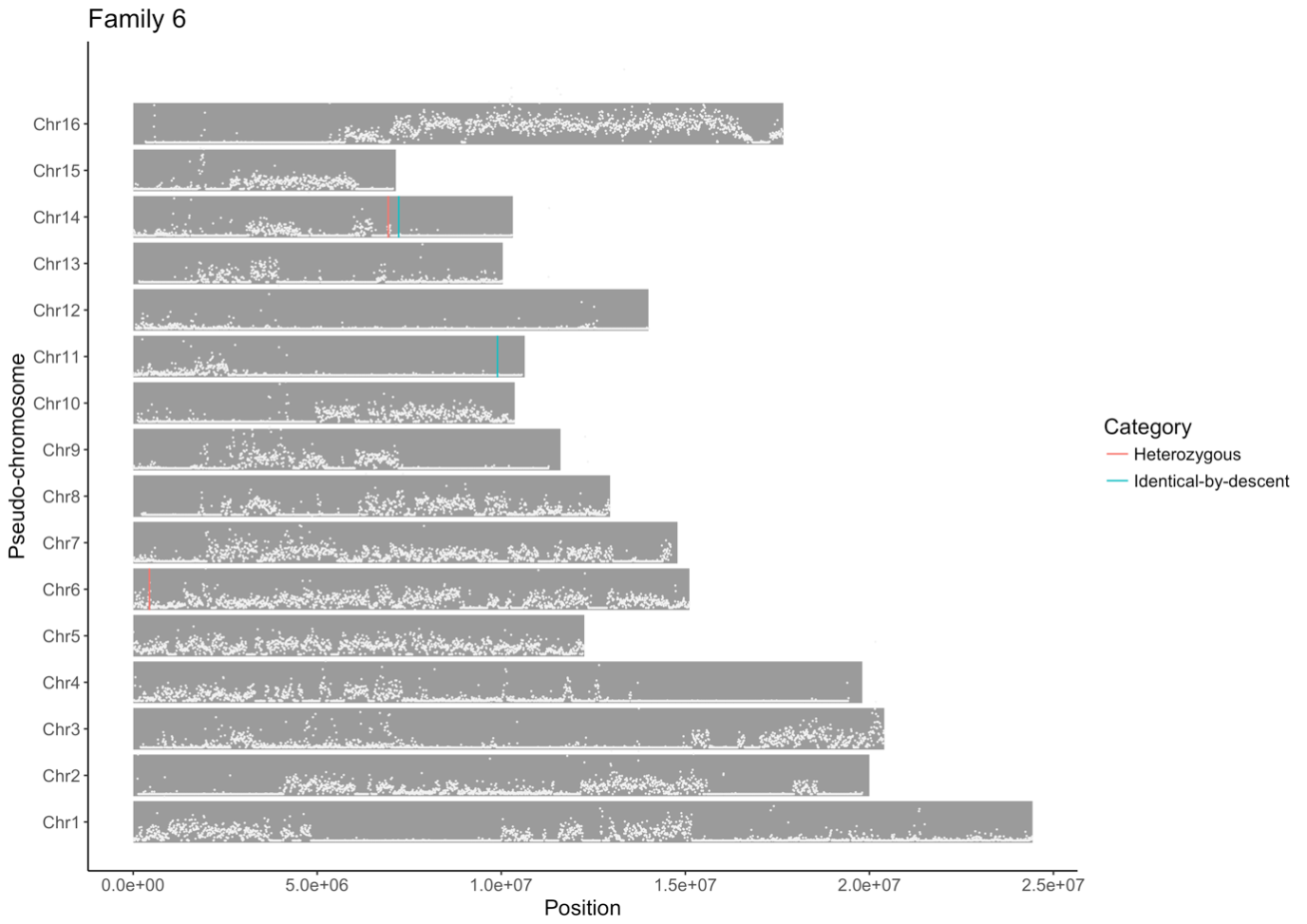


**Figure S9.**


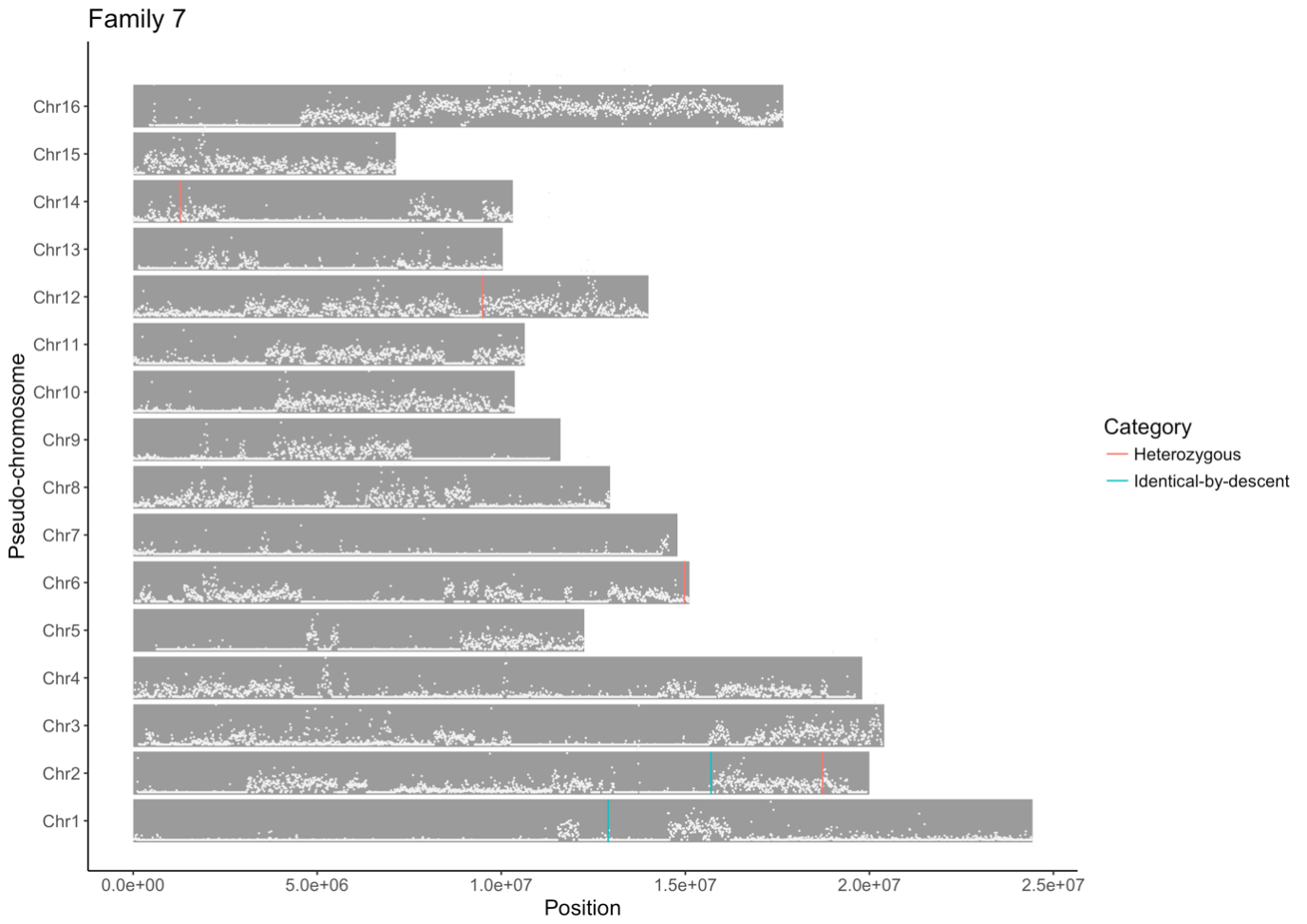


**Figure S10.**

**Figure S11. Detection of a somatic TE excision with low-coverage sequencing datasets.** We found a likely *Mariner-2_DF* somatic excision event in F7_b on pseudo-chromosome 2 at position 15,698,658 nt in an IBD segment in family 7 (A). Non-reference *Mariner-2_DF* insertion was annotated by ngs_te_mapper and was supported by BWA mapping in F7_B individual, where no reads crosses this insertion site. In contrast, the brother (F7_b) had a read pair (black rectangle) cross the insertion site indicating a potential somatic excision (B). This read mapping quality (mapq) is 60 indicating that it is not a mapping error.

**Figure S12. The *Mariner-2_DF* gene structure.** *Mariner-2_DF* is 1,322 bp and contains a 1,059 bp open reading frame (ORF). The ORF has HTH, DD34D, and YSPDLAP domains but lacks the WVPHL domain. The alignment shows the high sequence conservation of the transposase domains suggesting that all these fragments derived from the same element.

**Figure S13. High identity (>93%) of terminal inverted repeats (TIRs) for *Mariner-2_DF* in seven insect genomes.** TIR of *Drosophila erecta* and *Rhonius prolixus* are from the best hit of the genome copies. Other TIR sequences (xxx_gc) are from genome consensus sequences.

**Supplementary Tables**

**Table S2. Assembly statistics and quality control metrics for the fire ant germline transcriptomes.**

**Table S6. Mariner-2_DF family-specific non-reference insertions in *S. invicta*.**

**Table S7. Pairwise identity of *Mariner-2_DF* consensus sequences in each species.**

**Table S9. Estimation of the neutral mutation rate for 1951 common BUSCO genes: T=k/2r. T: divergence time. k: number of nucleotide substitutions per site. r: neutral mutation rate.**

**Supplementary Results**

**Completeness of germline transcriptome assembly**

We assessed the quality and completeness of our assemblies three ways. First, we determined the percentage of paired reads that mapped to the same transcript contig. Assemblies with >80% proper alignment are generally considered good (https://github.com/trinityrnaseq/trinityrnaseq/wiki/RNA-Seq-Read-Representation-by-Trinity-Assembly), and we obtained 97.11% (PO), 96.05% (MO), and 96.76% (MT). Second, we determined the percentage of assembled transcripts covering ≥80% of the corresponding protein length in the fire ant official gene set (GCF_000188075.1_Si_gnG_protein.faa). We found 71.90% (7,637/10,621) in PO, 69.54% (7,635/10,979) in MO, and 69.20% (7,159/10,345) in MT (Table S2). Our results are better than a recent *de novo* transcriptome study in a horned beetle (*Onthophagus sagittarius,* 50%) (Pespeni, et al. 2017) and comparable to the sugarcane (71.95%) (Hoang, et al. 2017). Third, we calculated a BUSCO score, which is a metric of gene set completeness based on conserved orthologous genes. Using the 2,675 arthropod BUSCO gene set, we found 2,285 (85.42%) complete BUSCO genes in PO, 2,286 (85.45%) in MO, and 2,231 (83.40%) in MT (Table S2). From these three tests, we conclude that the three independent transcriptomes have similar assembly qualities and are probably representative of the overall germline gene expression landscape.

**The remnants of *Mariner-2_DF* in three insects**

Inspection of *Mariner-2_DF* like sequences in *Drosophila yakuba*, *D. erecta*, and *R. prolixus* suggested that they were unlikely to be false positive hits. For two species, the TIR sequences have high identity to the *Mariner-2_DF* reference sequence: *D. erecta* (31/32, 96.88%) and *R. prolixus* (30/32, 93.75%) (Fig S11). Previous studies have shown that highly similar TIRs (≥84%) can be recognized by the same *mariner* transposase (Lampe, et al. 2001). Although we could not find TIR sequences in *D. yakuba*, we did find 6 fragments >100 bp with E-value < 1.20E-33 that were distributed across 4.2-23.7% (six together cover 31.98%) of the canonical *Mariner-2_DF* ORF, suggesting that they are also unlikely to be false positive hits. Thus, these fragments in *D. yakuba*, *D. erecta*, and *R. prolixus* are likely the remnants of *Mariner-2_DF* in the host genome, although we cannot exclude that full length sequences were missed due to the draft nature of these genomes.

**Newly determined consensus sequences for six highly expressed autonomous TEs determined in this study**

Of the 11 highly expressed TEs in the fire ant germline, the consensus sequence that we determined matched that of Repbase for five TEs (see also Table 1). For the remaining six, our consensus sequence differed from that in Repbase, and thus, are presented here. For *Mariner-35_SIn* we believe that Repbase version is incorrect. We find that the complete TE is a shorter internal sequence of 2080 bp and have thus renamed it here as *Mariner-35_SIn_curated_gc*. The terminal inverted repeat (TIR) sequences are underlined. The transposase open read frame (ORF) regions are shaded in grey. Sequence names are denoted with “gc” to indicate the genomic consensus sequence.

**TE consensus sequences**

>Mariner-4_AEc_SIn_gc

TTAGGAGTATAAATAAGTTTCCGCCGTTTCACAAAAAATGGCGCCACTAGTATGTTATGGTTGAAATTGTCTGTTGTAAAACGTTATATCGTAAGGTTGGACATCTGGCAACAACATAACCTTAAAATATTAGCGATTTTGTATCAGCATCATATATCTTTTTTCGTGCGAAAATGTCGAGTTTTGAGCCGAATAAGCGTCATTTGCGGGAGCTTTTGATTTACTTCTTTAATTTGAAGAAATCTGCAGCCGAGGCGCATCGATTGCTTGTAGAAGCATATGGTGAGGCTGCCTTAAGTGAGAGAAGTTGCCGTGAGTGGTTTCACAAGTTTAAGAACGGTGAATTTGACGTCGAAGACAAAGAACGTAGCGGAAGGCCGAAAGTGTACGAAGACGCGGAATTGGAAACATTATTAGATGAAGATTCGTGCCAAACGCAAGAAGCACTTGCACTTACATTAGGAGTGACTCAACCAGCAATTTCACATCGCTTAAAATCATTGGGAATGATTCAAAAACAAGGAAACTGGGTTCCATATGAACTGAAGCCGAGAAACGTTGAACGCCGATTTTTCACATGTGAAATGCTGCTTGCCAGGCATAAACGAAAGGGTTTTTTGCATCGTATAGTCACTGGTGATGAAAAATGGATCCACTACGATAACCCAAAGAAGAAGAAATCATGGGGACCACCTGGCCATGCTTCAACATCGACAGCCAAGCCGAACATTCATGGAAAAAAGCTCATGTTGTGTATTTGGTGGGATCAGCTTGGTGTCGTGTATTATGAGTTGCTCAAACCGAATGAAACCATTACTGGGGCTCTCTACCGAACACAATTGATGAGATTGAGCCGAGTACTCAAGGAAAAACGTGCCCACTACTACTCCAGACACGACAAAGTTATTCTTCTGCATGATAATGCTCGTCCACATGTTGCGGCGCCGGTCAAAACCTACCTGGAAACACTCAATTGGGAAGTTTTACCCCACCCGCCGTATTCACCAGACATTGCTCCTTCTGATTACTACCTGTTTCGATCGATGGCGCATGGCCTGTCTGAGCAACACTTCACATCATATGAAGATACAAAAAATTGGGTCGATTTGTGGATAGCTTCAAAAGATGAAGCATTCTTCCGACGCGGTATCCGTATGCTGCCAGAAAGATGGGAAAAAGTAGTGGCTAGCGATGGACAATACTTCGAATAAAACATTAGGTACCGTTCTTTCACAATAAATGCCCAATTTTTGATAAAAAACGGCGGAAACTTATTTATACTCCTAA

>Mariner-35_SIn_curated_gc

CAGTAATAAATTAAAATTATGTCATCACGGCAATATATTTTAGCTTCAATTTGTGGGACTATTACTTTACATAGAGACATAATTTTAATTAATTAATGAATGTAAATCTCTTTAATTACTTATTAAAATAATCTGTATTAGAAAATAATTTTAATTCGATTAATTTATAGATATGTTAACATTATCGGGCAAGCCAGTTTACTTTGTAATTGGCGTAAAACAATACTCTGAGAGAGGACCCGAATGAACGTCTGATTTTTCTTAAATTTCGTACAGTAATTTGTAACGTCAAAATATGAAGAAGTCTGTCACTTCTCCGAATGCCCCACATCCTTCTCGCTTGACGAGAGCCGAGAGAGAAAGCATGATACCATCTCTCGCGACGCGGCGTCAGGCCCCGGGAGGACGTATTTTGTTTTCTCTCTATCTCTTTTGCTCGTGCGTCTAAAGACAATAACGCATCTAACAAGAGACTAGCAAGAGACATCGCGAAATATGTTTTCTGCAGCTCATTTTCCGTGGTGTTCTTGCACAGTGCGGTGTATTCGCGAGTAAAATGCCTCAAAACTTGTCCTGCCAGAAGCGAGGTCGCATCGTCGGACAAGCTCAGGGCGGAAAATCAGTAGCAGAAATTGCTGCTGATATTCCGTGTTCTAAAAAAACTGTCCGACGATGGATACGACGTTTCGCAGAAGGCGGTGATGACGCTTTGAGTGATCATCGTCTGCATAATCGTCGTCCCCGTAAAATCGGTGCCGACGAAGTAGAGGCGATCGTCGCTGCGGCTGCTGATCGACCGTTCGGTAGCGTTCGGGAGTTCAGCAACGCAGTTGACGTGGATATATCTGAGAGGACCGTCCGGCGTCGTCTTAACGAAGCTGGGTATCGCTGTTACCGTCCAGCCTACAAAATTCCGCTGACTCCTGTTCATCGGGAGCAGCGAATCGCGTTCGCTTTGGAGAACAGTGTGACGAGCCGTGAGGATTGGGAGGCAACGATCTGGACCGATGAAAAGGTCTTTGTATCGTCTGCTGACCGCCAACCTCATGTATGGCGACTAAGAGATCAACGGTTGCATCCAAATCACGTTGTGCCCCTCCACAGAAGTGGAAGAATTTCATGCGCAATGTGGGGCTGGATTTCCGGCACTACTGTCGGCGAACTGGTGGAAACTCCCACACGCATGAACTCTCAGGACTATATTCGCATACTGGAAGAAGTTCTTCTTCCCTCAGTACGCGCAGTATATTCCGTAGAGGACATGCCGGTTATACGACTAGTGCAAGATAATTCCGCAGTCCACACTTCGCGTGAAACACAAACGTAGTTTCGGAATCATCCGGAGATACAATTAGTGAACTGGCCTGCTCGTTCACCAGATCTAAATTTAATCGAAAATGTTTGGGCCCAAATGGTTCGACGATGGGAACCAAGAAGGGAGAGGACGGTAGCAGCGTTAGTCAATCACGCGAGAGAAGTCTAGGAAGAACTTCGGTTCCACCCAGACTTCCTTGCAAACTTGATTGATTCGATACCTAATCGACTCAATCAAGTGATTGATCGGTCTGGATACTGGACAGACTATTAAAATATGCCGCGCTTGCACCACGATCGGCCCTTTTCAGGGCCAATAAAACTTCCATACCAAATAGGGACGCTTATAGAAGCGTTAGAATATGACAGGGCCCGACGCCGCGTCGCGAGAGATGGTATCATGCTTTCTCTCTCGGCTCTCGTCAAGCGAGAAGGATGTGGGGCATTCGGAGAAGTGACAGACTTCTTCATATTTTGACGTTACAAATTACTGTACAAAATTTAAGAAAAATCAGACGTTCATTCGGGTCCTCTCTCGGAGTATTGTTTTACGTCAATTACAAAGTAAACTGGCTTGCCCGATAATGTTAACATATCTATAAATTAATCGAATTAAAATTATTTTCTAATACAGATTATTTTAATAAGTAATTAAAGAGATTTACATTCATTAATTAATTAAAATTATGTCTCTATGTAAAGTAATAGTCCCACAAATTGAAGCTAAAATATATTGCCGTGATGACATAATTTTAATTTATTACTG

>Mariner-37_SIn_gc

CGAGGTGTGATCAAAAAGTAAGGTGACTTTTTGAATTTCGCGCGCTCTGTACACTCTAATTTCAAAAATTTTTTTTTTGTGTTGGTACACTCGTCACGATCATATGTTCACAGTTTTGACTATATAGCATGTGTTGTTTTTATGTGAGAGGCATAAAGGTTAGACTCGTGTTTGCGTGCTCGGCGATTTTTTGCTGTTGAAAATAATGGAGCAGAGAGTTTGTATTAATTTTTGTGTAAAAAATGGTATTAAGTGTTCAAAAACTCTTGAAATGTTGACAGTGGCGTACGGTGAGTCAACTTTGAGCAAAAAAAATGTTTATAAATGGTATAAGTTATTCCAAGAGGGCCGAGAAAATGTTAACGATGAACCTCGCTCTGGACGCCCCAGCACGTCAAAAACCGACGAAAATGTTCAGGAAGTGAAAGAAATTGTGTTGAAAAATCGTCGAATCACGATTAGAGAAATAGCTGATGATCTTAACATATCGTTTGGCTCATGCCAATCAATTTTAACGGATGTTTTGGGTATGACACGTGTGTCAGCGAAATTCGTTCCAAAACTGCTTAATTTTGATCAGAAGCAGCGTCGCATGAACATCGCCCAAGACATGTTGAACGACGTCAATGATGATCCTGATCTGCTCAAAAGGGTTATAACTGGTGACGAAACATGGGTATATGGTTATGACGTCGAAACCAAAGCCCAATCATCCCAGTGGAAGAGCCCAGGAGAGCCAAGACCGAAAAAGGCACGCCAAGTTCGTTCGAATGTGAAGGTTTTGCTCACAGTTTTCTTTGATTACCATGGCGTTGTGCATCAAGAATTCCTACCACAAGGTCGTACGGTAAACAAGGAGTATTACCTTGAGGTTATGCGGCGTTTGCGTGAATCAATAAGAAAAAAACGTCCGGAAGTGTGGAAAGAAAATTCATGGATTCTGCACCATGATAATGCACCTGCGCACACGTCGTTACTAGTGAGTACTTTTTTGGCCAAAAACAATACTATCATCATGCCTCAGCCACCGTATTCACCAGACTTGGCCCCCTGCGACTTTTTCCTCTTCCCAAAATTGAAAAGGCCTATGAAAGGACGAAGATTTGCGACGATTGAGGAGATTAAGGCTGCATCGCTGGAGGAGCTCAAGGCAATACCCAAAAGTGCATTTCAGAAATGTTTTGACGACTGGAAAAAGCGCTGGCACAAATGCATTGTATCAGAGGGGGATTATTTTGAAGGGGATAACATAATTTTGGATGAATAAATGAATATTTTTTTATAAAAATGAAAAGTCACCTTACTTTTTGATCACACCTCG

>Mariner-2_DF_SIn_gc

CGAGGTGTGTTCAAAAAGTATCGCGAATTTTGAATTTTCGCGGGTTACGTATATTCGAATTTCGATCTTTTTGTGGCGTTATGTTGGTACTCATGTCTCTCACTTATGCCGACAAGCTCGGCCATTTTGAATGTTCACTTAATTGTTGACAGCTGCTTTGCTTGCACGTGTTTTGGATCGTCTTCGATTTTTACCTATTCAAAAAAATGGATCAAAGAACCTGTATCAAATTTTGTGTGAAAAACGAAATTAAGTGCGCGGATGCATTCCGAATGTTGACTGTGGCATACGGAGAAGCTACCTTGGACCGAAGCAACGTTTATCGGTGGTACAAAATGTTCTCAGAAGGCCGAGAAGATGTGAACGACGAAGAGCGTGCCGGACGCCCGAGCACTTCAACAACAGACGAAAAAATTAATGAAGTGGAGAAAATGGTATTGGCCAATCGTCGAATCACCGTTAGAGAAGTTGCTGAGGACCTAAACATATCGATTGGCTCGTGCCATTCGATTTTTATCAATGATTTGGGCATGAGACGGGTCGCCGCGAAATTCGTACCAAAATTGCTCAATTGCGACCAAAAACAGCATCGCATGAACATTGCTAATGAGATGTTGGACTCTGTCCGCGACGACCCAAATTTGCTCCAGAGGGTCATAACTGGTGACGAATCGTGGGTTTATGGTTATGACGTGGAAACCAAAGCTCAATCATCTCAATGGAAGCTGCCGCACGAACCAAGACCGAAAAAAGCGCGCCAAGTTCGGTCGAATGTGAAAGTTTTGCTGACAGTTTTCTTCGATTGCAGGGGCGTGGTGCATCATGAGTTCTTGCCACAGGGTAGAACGGTCAATAAGGAATATTACCTGCAAGTTATGCGCAATTTGCGCGAAGCAATCCGCCAGAAACGCCCGGATTTGTGGAAGAACAAAAATTGGCTTTTGCACCACGATAACGCCCCTGCTCACACATCGTTGCTTGTGCGCGACTTTTTGGCCAAAAACAACACACTAATGATGCCGCAGCCACCGTATTCCCCAGATCTGGCCCCCTGTGACTTTTTCTTGTTCCCTAAACTGAAGAGGCCCATGAAAGGACGACGTTACGCTACGCTTGACGAGATAAAGACGGCATCGAAGGAGGAGCTGAACAAGATAAAAAAAAATGATTTTTTGAAGTGCTTCGAAGATTGGAAAAACCGTTGGCACAAGTGTATAATATCTCATGGGGATTACTTTGAAGGGGACAAAATAGATATTCATGAATAAATAAATAATTTTTGAAAAAACACAAAATTCGCGATACTTTTTGAACACACCTCG

>Mariner-30_SIn_gc

TTAGGAGTACAAATTGAAAACCGCCGTTTTCCAGTAGATGGCGCCAGCGGTAAATGCTGGCGCCAAACGTTAGATCAAAAATTTTAAATGTAAGGTTGGGCATCTGGCAACAATTCCTTATCATTGCAGTTGTGAATTTTTATCGGCGTTATATATTTTTTTGTGATCGAAAATGTCGAAATTTGTGCCCAATAAGCGTCATTTGCGGGAAGTTTTGCTTTTTGCCTTCAATTCGAAAAAATCTGCGGCTGAGGCGCGTCGAATGATTGTAAAAACTTATGGTGAGGCTTCCATTAGTGAAAGAACGTGTCGAGAATGGTTCCAACGCTTCAAAAGTGGTGATTTCGGCGTAGAAGACAAGGAGCGTCCCGGACAGGTGAAAAAGTTTGAAGACGCACAATTGGAAACATTACTGAATGAAGATTCATCTCAAACGCAACAGGAGCTTGCAGATTCATTGGGCGTGACTCAACAAGCTATTTCACATCGTTTGAAAACCATGGGAATGATCCAAAAGCATGGACACTGGGTGCCATACGAATTAAAGCCGAGAGACGTCGAACGGCGTTTTTTCGCGTGCGAACAGCTGCTCCAACGGCAAAAACGGAAGGGTTTTCTGCATCGTATTGTAACCGGCGATGAAAAGTGGATCCATTATGATAATCCAAAGCGAAAAAAATCGTGGGGCTACCGCGGCCATGCATCAACATCGACGGCCAAGCCAAACATCCATGGCTCGAAGCTCATGCTGTGTATTTGGTGGGACCAGCTCGGCGTGATTTATTATGAGCTGTTGCAACCGGGTGAAACCATCACAGGAGCTCGCTACCGAACACAACTAATGCGTTTGAGCCGAGCATTGCAGGAAAAACGGCCACAATACGAGCAAAGATACGAAAAAGTGATGTTGCTGCACGACAACGCTCGGCCACGTGTTGCTCAGGTCGTTAAAACCTATCTGGAAACATTGAAATGGGACGTCTTACCTCATCCGCCGTATTCTCCTGACATCGCCCCTTCAGATTACCACTTGTTCCGATCAATGGCGCATGGCCTGGCTGAGCAGCACTTCCATTATTACGAAGAGGCCAAAAACTGGGTCGATTCGTGGATCGCCGCAAAAGACGAGCAGTTTTTTCGACGCGGGATTCGTATGCTGCCCGAAAGGTGGGAGAAAGTAGTGGCCAGCGATGGACAATACTTTCAAGAATAAGTATGTAACCATTTTTCAACAATCAATCCTCAAATTTTGACAAAAAACGGCGGTTTTCAATTTGTACTCCTAA

>Mariner-50_HSal_SIn_gc

CTACTGTGTTCAAAAAGTAAAGTGAATTTTTAATTTAAACTTCCCGCGCTAATCGATTCGAGCAAACTGTTTTATTTTTATGTTGGTACTACTGTTAGTGACATCTGTGCCAAATTTCATGTGAATGTCATCATTAGTCATATAGTTACGCTTGTGTTTTCTAAACGACTAAAAGTGATTTTGGCGATTTTTACAATGTCTGATTTTGCTGAGCAAAGAAGTGCCATTAAATTTTGTTTGCGGAATGAAATTTCGGCTGCGGAAACGTGCAGAATGTTGCAGAGGGCCTTCGGTGAATCGACTATGTCGCAGAAAAATGTTTATAAGTGGTACAAAGACTTCAAAGAAGGCAGAGAACGTGCTGATGACTTGGAACGCTCCGGACGACCATCAACGTCAACTGATGAGCAACACGTAAAGAAAATCAAGGAATTGGTGCTCGAAAATCGTCGATTGACAATTAGAGACCTTGTCGATATGGTTGGCATTTCAATTGGATCAGTTCAAACAATTTTGAAGGATCATTTGGGCCTTAGAAAAGTCAAATCTCGTTTGGTGCCAAAAACACTCAATTTCCTCGAAAAAGAGCGTCGCGTTAAAGTTTGTGAAGAGATGATTTCTGACTATCAGGACGTCTACGAACGCATTATTACGGGAGATGAGACTTGGATCTATGCTTATGATCCGGAAACAACCGACCAATCGAGTGAATATCGTGCTAAAGGCGAGGCAAGACCAAAAAAATCGCGCCAAAGTCGCTCAAAAATCAAGGTTATGTTGACAGTTTTCTTCGACTATCGTGGTGTTGTGCATTATGAGTTCCTTCCGCCGGGCCAAACTGTCAACAAGGAATATTATTTGAGTGTTATGCGTCGTTTGCGTGAAGCTATTCGCAAGAAGAGGCCGGATTTATGGAAAGACAACTCCTGGTTTTTGCATCACGATAATGCGCCGTCTCACACGGCATTGATTCTTCGTGAGTTTTTCGCCAAAAATTCCACTCATATCGTTCCGCAACCGCCTTATTCACCTGACTTAGCTCCGTGTGACTTTTGGCTATTTCCCAAACTCAAAAGACCGCTCCGGGGACACCGCTTTGAATCTATTGAAGAGATTCAACGTGAATCGCTGCGCGCTTTGAAGGCTATACCGGGAAACGACTTTTTGGCATGCTTCGAGGAATGGAAAAAACGTTGGCATAAGTGCATTGTATCGGAGGGGGAATACTTCGAAGGGGATGAAATTGATTTGGATGAATAAATAAAGATTTTTCATTTTATAACAAAATTCACCTTACTTTTTGAACACAGTAG

>Mariner-12_LHu_SIn_gc

TTAGGTGTTGGAATTAATTCGTAGCGTTTTACGAGAGATGGCGGGGATACTACAGTTTTCTGTTTTTACCGTCAGAAGCAAATTTATCTTAAATCAGTTGTTAGGTTAGGTGTTATTTTTACGACATCTTACTTTCACTTGTTAATAACATCAAGTTATAATTATTTAAATATGTCTAAATTTATACCAAATAAAGTGTTTTTGCGGGGAGTTTTATTACATTACTTTCATATGAATAAAACTGCTGCGGAAAGCCATAGGATTTTAGAAGAAGTTTACGGTGAGCAAGCTTTAGCTGAACAAACGTGTCGGAAGTGGTTTGCGCGATTCAAGAGCGGTGATTTCGACTTAGAAGACAAGGAACGACCTGGACAGCCAAAAAAGTTTGAAGACAAAGAATTGGAGACATTACTTGAGGAGAACTCAACTCAAACGCTCGAAGAGTTGTCCACATCGTTGGGGGTTGACTTTTCAACAGTGGGAAAACGACTTAAAGCGTTAAGGATGATCCAAAAGGAAGGACATTGGATCCCATACGAATTAAAGCCGAGGGACGTTGAAAGGCGTTTTTTGACTTGCGAGTTACTGCTTCAACGGTACAAAAGAAAAAGTTTTTTACATCGGATTGTTACGGGTGATGAAAAGTGGATATATTACGATAACCCCAAGCGCAAAAAATCATGGGTTAAGCCTGGACAACCATCATCATCAGTACCAAAACGTAATATTCATGGATCTAAGCTTCTTCTTTGCATTTGGTGGGATCAACGGGGTGTGGTTTACTACGAACTGCTGAATCCGAATGAAACCATCACTGGGGATCGTTACCGACTGCAATTGATGCGCTTGAGTCGGGCATTGAAGGAGAAACGGCCGGAATGGAGCGAAAGGCACGACAAAATTATTTTGCTGCACGACAATGCTAGACCGCACGTCGCAAAGCCAGTGCAAACGTACCTGCAAACACTGAAATGGGAAGTCCTAACCCACCCGCCGTATTCTCCAGATATTGCTCCTTCGGATTATCACTTGTTCCGGTCGATGGCCCATGGTTTGTCCGCGGAGAAATTTACTTCTTATGAAGAATGCAAAACTTGGATTGATTCATGGATCGCCTCTAAGGACGAAGCGTTTTTTCGACGGGGTATCCGTCTCTTACCCGAAAGATGGGAAAAAGTTGTGGCAAGCGATGGAAAATATTTTTAGTAATGTATTTTTATATTTAAACTTAGAATAAATCGTTGTTTCTCTCTAAAAAAACGCTACGAATTAATTCCAACACCTAA

**Supplementary Methods**

**Analysis command line**

#### Transcriptome *De novo* assembly (using Monogyne ovary as an example)

## 1 ## Read quality control

### 1-1 ### Remove non-random bases, head 17 bp and 5 bp tail

cutadapt --cut 17 -o DeHead_L007_R1.fastq ST-EG02_TGACCA_L007_R1.fastq

cutadapt --cut 17 -o DeHead_L007_R2.fastq ST-EG02_TGACCA_L007_R2.fastq

cutadapt --cut -5 -o DeBoth_L007_R1.fastq DeHead_L007_R1.fastq

cutadapt --cut -5 -o DeBoth_L007_R2.fastq DeHead_L007_R2.fastq

### 1-2 ### Remove adapters

cutadapt \

-a GATCGGAAGAGCACACGTCTGAACTCCAGTCACTGACCAATCTCGTATGCCGTCTTCTGCTTG \

-A GATCGGAAGAGCACACGTCTGAACTCCAGTCACTGACCAATCTCGTATGCCGTCTTCTGCTTG \

-q 28 -m 50 -o MVQ_deA_R1.fastq -p MVQ_deA_R2.fastq DeHead_L007_R1.fastq \

DeHead_L007_R2.fastq

## 2 ## *de novo* assembly with Trinity

Trinity --seqType fq --max_memory 50G --left MVQ_deA_R1.fastq --right \

MVQ_deA_R2.fastq --CPU 14 --min_contig_length 305 --verbose

## 3 ## Transcriptome assembly quality assessment

### 3-1 ### Read representation

bowtie2 --local --no-unal -x Trinity.fasta -q -1 MVQ_deA_R1.fastq \

-2 MVQ_deA_R2.fastq | samtools view -Sb - | samtools sort –no - - \

> bowtie2.nameSorted.bam

SAM_nameSorted_to_uniq_count_stats.pl bowtie2.nameSorted.bam

### 3-2 ### Representation of full-length protein-coding genes

makeblastdb -in GCF_000188075.1_Si_gnG_protein.faa -dbtype prot

blastx -query Trinity.fasta -db GCF_000188075.1_Si_gnG_protein.faa \

–out MVQ_blastx.outfmt6 -evalue 1e-20 -num_threads 6 \

-max_target_seqs 1 -outfmt 6

analyze_blastPlus_topHit_coverage.pl MVQ_blastx.outfmt6 Trinity.fasta \

GCF_000188075.1_Si_gnG_protein.faa

### 3-3 ### Using BUSCO to examine assembly completeness

python BUSCO_v1.22.py -in Trinity.fasta -o MVQ_BUS -m trans \

-l arthropoda -c 6

## 4 ##Transcript Quantification

### 4-1 ### Removed rRNA contamination

sortmerna --ref \

./rfam-5.8s-database-id98.fasta,./rfam-5.8s-database-id98-db: \

./rfam-5s-database-id98.fasta,./rfam-5s-database-id98-db: \

./silva-arc-16s-id95.fasta,./silva-arc-16s-id95-db: \

./silva-arc-23s-id98.fasta,./silva-arc-23s-id98-db: \

./silva-bac-16s-id90.fasta,./silva-bac-16s-id90-db: \

./silva-bac-23s-id98.fasta,./silva-bac-23s-id98-db: \

./silva-euk-18s-id95.fasta,./silva-euk-18s-id95-db: \

./silva-euk-28s-id98.fasta,./silva-euk-28s-id98-db \

--reads MVQ_merge.fastq \

--aligned SortMeRNA_out_silva_MVQ.fastq --other No_rRNA_silva_MVQ.fastq \

--fastx --paired_in -m 4096 --log –v

### 4-2 ### Estimating transcript abundance via RSEM method

align_and_estimate_abundance.pl --transcripts Trinity.fasta \

--seqType fq \

--left MVQ_deA_R1.fastq --right MVQ_deA_R2.fastq --est_method RSEM \

--aln_method bowtie2 --thread_count 3 --trinity_mode --prep_reference \

-- output_dir Estimate_MVQrepTE_rsem

#### Variant calling (using F1_B as an example)

## 1 ## Read quality control

cutadapt -a XXXXXXX -A XXXXXXX --cut 10 -q 28 -m 50 -o F1_B_R1.fastq –p \

F1_B_R2.fastq SRR620242_1.fastq SRR620242_2.fastq

| SRA file | Family | Social chromosome |
| --- | --- | --- |
| SRR620242 | F1 | SB |
| SRR620546 | F1 | Sb |
| SRR620547 | F2 | SB |
| SRR620564 | F2 | Sb |
| SRR620565 | F3 | SB |
| SRR620566 | F3 | Sb |
| SRR620567 | F4 | SB |
| SRR620568 | F4 | Sb |
| SRR620570 | F5 | SB |
| SRR620571 | F5 | Sb |
| SRR620572 | F6 | SB |
| SRR620573 | F6 | Sb |
| SRR620574 | F7 | SB |
| SRR620577 | F7 | Sb |

## 2 ## Generate index file

java -jar /home/volans/picard-tools-2.1.1/picard.jar \ CreateSequenceDictionary R=PM2_chr_masked.fasta O=PM2_chr_masked.dict

bwa index PM2_chr_masked.fasta

## 3 ## Mapping reads via BWA

bwa mem -t 16 PM2_chr_masked.fasta F1_B_R1.fastq F1_B_R2.fastq > \

F1_B_bwa.sam

## 4 ## Convert “.sam” to “.bam” file

java -jar /home/volans/picard-tools-2.1.1/picard.jar SamFormatConverter \

I=F1_B_bwa.sam O=F1_B_bwa.bam

## 5 ## Sort bam file

java -jar /home/volans/picard-tools-2.1.1/picard.jar SortSam \

SORT_ORDER=coordinate I=F1_B_bwa.bam O=F1_B_bwa_sort170317.bam

## 6 ## Add group information

java -jar /home/volans/picard-tools-2.1.1/picard.jar \

AddOrReplaceReadGroups \

INPUT=F1_B_bwa_sort170317.bam OUTPUT=F1_B_bwa_sort170317_addRG.bam \

RGID=1 RGLB=LGC-GK01_575bp RGPL=illumina RGPU=ACAGTG RGSM=F1_B

## 7 ## Mark duplicate reads

java -jar /home/volans/picard-tools-2.1.1/picard.jar MarkDuplicates \

I=F1_B_bwa_sort170317_addRG.bam O= Mdup_F1_B_bwa_sort170317_addRG.bam \

M= F1_B_bwa_sort170317_addRG_Mdup_metrics.txt

## 8 ## Sort “.bam” file in coordinate

java -jar /home/volans/picard-tools-2.1.1/picard.jar SortSam \

I=Mdup_F1_B_bwa_sort170317_addRG.bam \

O=Sort2_Mdup_F1_B_bwa_sort170317_addRG.bam SORT_ORDER=coordinate

## 9 ## Generates a BAM index ".bai" file

java -jar /home/volans/picard-tools-2.1.1/picard.jar BuildBamIndex \

I=Sort2_Mdup_F1_B_bwa_sort170317_addRG.bam

## 10 ## Create realignment targets

java -jar /home/volans/GenomeAnalysisTK.jar -T RealignerTargetCreator \

-R PM2_chr_masked.fasta -I Sort2_Mdup_F1_B_bwa_sort170317_addRG.bam \

-o Sort2_Mdup_F1_B_bwa_sort170317_addRG.intervals

## 11 ## Indel realignment

java -jar /home/volans/GenomeAnalysisTK.jar -T IndelRealigner –R \

PM2_chr_masked.fasta -I Sort2_Mdup_F1_B_bwa_sort170317_addRG.bam \

-targetIntervals Sort2_Mdup_F1_B_bwa_sort170317_addRG.intervals \

-o F1_B_bwa_realigned.bam

## 12 ## Call variants (UnifiedGenotyper)

java -jar /home/volans/GenomeAnalysisTK.jar -T UnifiedGenotyper \

-R PM2_chr_masked.fasta \

-I F1_B_bwa_realigned.bam \

-I f1b_bwa_realigned.bam \

--sample_ploidy 1 -o NatureMaleF1.snps.raw.vcf

## 13 ## Filter homozygous (false positive) SNP call

vcftools --vcf NatureMaleF1.snps.raw.vcf \

--maf 0.4 --recode --out NatureMaleF1_maf04

## 14 ## Calculate the SNP density

vcftools --vcf NatureMaleF1_maf04.recode.vcf \

--SNPdensity 10000 --out NatureMaleF1_maf04_SNPdent10k

**Supplementary References**

Hoang NV, et al. 2017. A survey of the complex transcriptome from the highly polyploid sugarcane genome using full-length isoform sequencing and de novo assembly from short read sequencing. BMC genomics 18: 395.

Lampe DJ, Walden KK, Robertson HM 2001. Loss of transposase-DNA interaction may underlie the divergence of mariner family transposable elements and the ability of more than one mariner to occupy the same genome. Molecular biology and evolution 18: 954-961.

Pespeni MH, Ladner JT, Moczek AP 2017. Signals of selection in conditionally expressed genes in the diversification of three horned beetle species. Journal of Evolutionary Biology.
